# Materials Perspectives of Integrated Plasmonic Biosensors

**DOI:** 10.3390/ma15207289

**Published:** 2022-10-18

**Authors:** Ayman Negm, Matiar M. R. Howlader, Ilya Belyakov, Mohamed Bakr, Shirook Ali, Mehrdad Irannejad, Mustafa Yavuz

**Affiliations:** 1 Department of Electrical and Computer Engineering, McMaster University, Hamilton, ON L8S 4K1, Canada; 2 Department of Electronics and Communications Engineering, Cairo University, Giza 12613, Egypt; 3 Waterloo Institute for Nanotechnology, University of Waterloo, Waterloo, ON N2L 3G1, Canada; 4 School of Mechanical and Electrical Engineering Technology, Sheridan College, Brampton, ON L6Y 5H9, Canada; 5 OZ Optics Ltd., Ottawa, ON K0A 1L0, Canada

**Keywords:** plasmonics, plasmon resonance, plasmonic biosensing, plasmonic materials, substrates, 2D nanomaterials, TMD, graphene, point-of-care devices, LSPR, SPR

## Abstract

With the growing need for portable, compact, low-cost, and efficient biosensors, plasmonic materials hold the promise to meet this need owing to their label-free sensitivity and deep light–matter interaction that can go beyond the diffraction limit of light. In this review, we shed light on the main physical aspects of plasmonic interactions, highlight mainstream and future plasmonic materials including their merits and shortcomings, describe the backbone substrates for building plasmonic biosensors, and conclude with a brief discussion of the factors affecting plasmonic biosensing mechanisms. To do so, we first observe that 2D materials such as graphene and transition metal dichalcogenides play a major role in enhancing the sensitivity of nanoparticle-based plasmonic biosensors. Then, we identify that titanium nitride is a promising candidate for integrated applications with performance comparable to that of gold. Our study highlights the emerging role of polymer substrates in the design of future wearable and point-of-care devices. Finally, we summarize some technical and economic challenges that should be addressed for the mass adoption of plasmonic biosensors. We believe this review will be a guide in advancing the implementation of plasmonics-based integrated biosensors.

## 1. Introduction

Plasmons are the collective oscillation of charges due to the interaction between an electromagnetic (EM) wave and the free electrons at the metal/dielectric interface [1,2]. The energy in the incident wave is transferred in the form of a transverse-magnetic wave propagating along the interface while decaying in metal and dielectric [3,4] In the case of a nanostructured configuration, the energy is transferred in the form of a highly confined field within voids or grooves called “hot spots” [5,6]. This coupled energy can be manipulated within dimensions below the diffraction limit [7,8].

Plasmonic resonance shifts can be tuned and enhanced by varying the size and shape of plasmonic materials to achieve the desired level of sensitivity [9,10]. Two major concepts for biosensing applications are nanofilm- and nanoparticle-based plasmonic materials, which also have important optical properties for these applications [11]. Major plasmonic materials include metals such as gold (Au), silver (Ag), copper (Cu), and aluminum (Al), doped semiconductors such as aluminum-doped zinc oxide and indium-doped tin oxide, transition metal dichalcogenides (TMDs), and ceramics such as titanium nitride. In comparison to metals, which are in general good plasmonic materials due to the abundant free electrons [12], semiconductors have the ability to form surface plasmons via doping [13] Moreover, metals are not chemically stable, suffer from high ohmic losses, and their conductivities are not tunable. Two-dimensional (2D) nanomaterials, such as graphene and TMDs can accommodate surface plasmons [14,15]. Graphene plasmonics operate from terahertz to mid-infrared frequencies for devices such as optical modulators, photodetectors, and biosensors [16]. Meanwhile, TMDs such as MoS_2_ and WS_2_ operate in visible range which can be used in biosensing and photodetector applications [17].

There are two main plasmonic modes for applications such as biosensing, localized surface plasmon resonance (LSPR) or propagated surface plasmon resonance (SPR) modes. LSPR sensing is more suitable for portable and wearable biosensing needs such as in point-of-care (PoC) applications due to its ease of control, and multiple, parallel sensing capability, while using miniaturized excitation and detection devices. Different physical approaches, including electric, magnetic, toroidal, Fano, and Fabry–Perot modes, can be used for the excitation of plasmonic resonance in LSPR [18] Even though the sensitivity towards biomolecular binding events is similar in LSPR and SPR, the SPR has higher refractive index sensitivity. On the other hand, the SPR mode requires complex equipment and a large setup which is not suitable for PoC applications. With the goal of targeting future PoC biosensors, the study in [19] focused on promising technologies such as chiral, magneto, and quantum plasmonics. The remaining challenges include the material losses, lack of selectivity of the sensing device to different analytes, particle control in fluids, and localization of target analytes within the device hot spots.

Nanofabrication technologies provide a wide range of plasmonic nanomaterials, structures, and components, and their robust integration into a common platform. Examples include top-down techniques such as EBL and FIB that provide accurate control over the size and shape [20]. Bottom-up techniques such as nanospheres lithography and chemical vapor deposition provide lower cost and higher throughput at the expense of lower resolution [21]. Additionally, techniques such as 3D printing and DNA assembly can be used for microfluidic-based future biosensors [22]. Advances in nanofabrication technologies would offer a path towards achieving a small footprint and integrated systems, such as self-powered wearable biosensing applications. Unfortunately, to the best of our knowledge, while there is a considerable amount of work in developing discrete plasmonic devices, the integration of diverse plasmonic materials on a common substrate to create plasmonic systems has not yet been fully realized. The system-level integration offers several advantages, such as portability, specificity to different analytes, automation, high throughput, reduced sensing time, and cost reduction [23]. Such integration is critical to combining different functionalities under various operating conditions, such as wearable biosensors [24].

In this article, we review different modes of plasmonic biosensing, and the physics behind them, as well as covering materials for the substrate and sensors. As more focus is currently being paid towards integrated and portable biosensors, our review provides materials perspectives of plasmonic biosensors with the aim of summarizing the key merits of plasmonic materials for the integration of biosensors, as well as identifying the main challenges that should be addressed towards this goal. The paper is organized as follows: Section 2 explains the physical aspects of plasmonic behavior for both SPR and LSPT modes, followed by the functional benefits of plasmonic biosensors in Section 3. In Section 4, we cover the materials used for active (plasmonic) material and substrate. Finally, in Section 5, the future perspectives of plasmonic biosensors with conclusions and suggested future research directions are provided.

## 2. Plasmonic Operation

SPR and LSPR are the two modes for plasmonic operation [25,26]. In the propagating SPR mode, the incident EM wave excitation couples to propagating modes at the interfaces between one or more metal/dielectric boundary. The key components operating in this mode include waveguides, couplers, and splitters [27]. Compared to SPR, the incident EM excitation in LSPR mode creates localized confinement of charges at the metal/dielectric interface. Examples of the LSPR components include nanoholes, nanowires, nanorods, and nanoparticles [28]. The resonance profile and frequency depend on the plasmonic material employed and the refractive index of the surrounding media [29]. The changes in this refractive index precisely shift the resonance frequency, which can be defined as sensitivity in plasmonic sensor applications [29].

(1) $$S_{\lambda }=\frac{\delta \lambda }{\delta n}$$

where $\delta \lambda \;\left(\mathrm{nm}\right)$ and $\delta n\;\left(RIU\left(refractive\;index\;unit\right)\right)$ are the shift of resonance wavelength and change of the refractive index in surrounding media, respectively. In the next two subsections, the physical behavior of both SPR and LSPR modes will be discussed.

### 2.1. Propagating Mode

**Figure 1:**
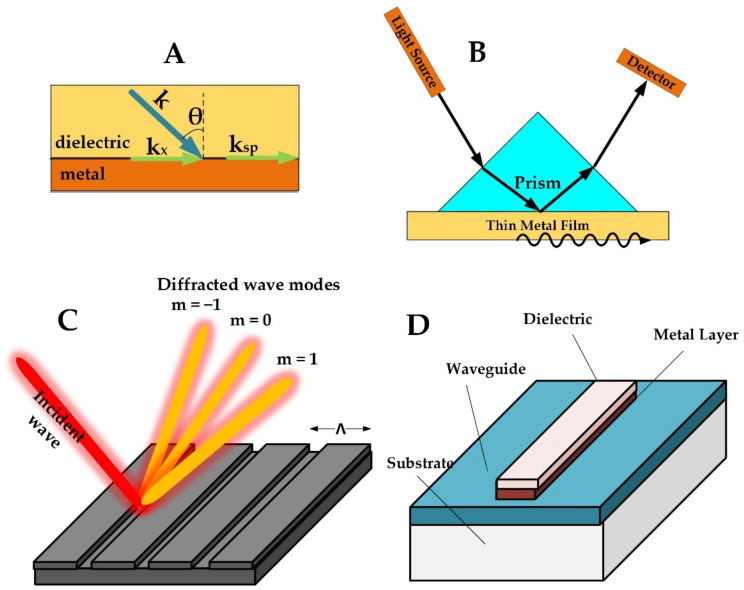
(**A**) Geometry for surface plasmon wave. (**B**) The basic Kretschmann configuration for biosensing. (**C**) Diffraction grating configuration. (**D**) Waveguide configuration.

Figure 1 shows the basic structure for attaining a propagating plasmonic wave. In this mode, coupling between the incident wave and the plasmonic waves on the metal surface is essential. This coupling condition can be deduced by matching the propagation constant of a plasmonic wave to the propagation constant supported by the waveguide along the interface [11]. The electrical permittivity of the metal can be used to characterize its charge carriers, which can be defined as:

(2) $$\varepsilon_{m}=\varepsilon_{\mathrm{mr}}+{i\varepsilon }_{\mathrm{mi}},$$

where $\varepsilon_{\mathrm{mr}}$ and $\varepsilon_{\mathrm{mi}}$ are the real and imaginary components of the electrical permittivity, respectively. The value of the real part indicates the strength of polarization in the metal, while the value of the imaginary part indicates the losses encountered due to polarization in the metal [30]. The dispersion relation for the plasmonic wave propagating at the interface between the metal and the dielectric is given by [31,32]

(3) $$k_{\mathrm{sp}}=\frac{\omega }{c}{\left(\frac{1}{\varepsilon_{d}}+\frac{1}{\varepsilon_{m}}\right)}^{-0.5}$$

where ω is the angular frequency, c is the speed of light in vacuum, and $\varepsilon_{d}$ is the permittivity of the dielectric medium. For a surface plasmon wave to propagate along the surface, $k_{\mathrm{sp}}$ must have a real component, which implies that $\varepsilon_{\mathrm{mr}}$ must be negative with a magnitude greater than $\varepsilon_{d}$. Metals such as gold, silver, and aluminum have negative $\varepsilon_{\mathrm{mr}}$ in the visible and near infrared regions [11]. For an incident wave of wave number k, the projection of the wave along the interface is given by:

(4) $$k_{x}=\frac{\omega }{c}\sqrt{\varepsilon_{d}\;}\sin\theta ,$$

where θ is the angle of incidence of the exciting wave. If the real part of the wave number defined in Equation (3) is matched to the wave number defined in Equation (4), coupling takes place between the incident wave and the plasmonic mode of the interface and a surface-propagating wave is obtained. The matching condition implies that the wave is propagating along the interface and decaying along the directions perpendicular to it. Due to the imaginary part of the metal’s permittivity, the surface plasmon wave propagation is attenuated along the interface [11]. The propagation length defines the feature size of the surface supporting the wave [33,34].

Different types of configurations can be used to excite propagating surface plasmonic waves, including the Kretschmann configuration, the diffraction grating configuration, and the waveguide-coupled configuration [35]. Figure 1B shows the basic structure of the Kretschmann configuration. A light source is used for illuminating the plasmonic surface at an angle larger than the critical angle of incidence to maintain total reflection [36], and a detector is used for analyzing the spectrum reflected from the surface. A prism is placed above the metal surface to compensate for the difference in momentum between the incident wave and the plasmonic mode along the metal surface. This configuration is a reliable setup for obtaining an efficient biosensor with high sensitivity. Utilizing this configuration, different interrogation methods can be used for the detection of analytes that are bound to the metal surface such as the wavelength, the phase, or the intensity [36]. When maximum coupling occurs, optimal energy transfer takes place at the interface, indicated by minimum reflectivity measured by the detector [32]. Different analytes attached to the thin metallic film can then be sensed by tracking the changes in these coupling conditions [37].

Although the Kretschmann configuration is widely used, it has some disadvantages. The structure has large optical components such as the light source and the spectrometer, in addition to its high cost [38]. These factors make it impractical for integrated designs [39]. Moreover, it is not suitable for multiplexed sensing of multiple analytes simultaneously [40]. One of the best efforts to miniaturize the configuration was demonstrated in [41] where the whole setup was packaged in a volume of 700 mL, which still is not miniaturized enough for PoC applications.

Surface plasmon waves can also be excited using a diffraction grating as shown in Figure 1C. In this configuration, the surface of the plasmonic material is shaped in the form of a periodic diffraction grating. When an incident wave falls on the grating surface, different diffraction modes arise, and coupling takes place when the diffraction mode matches the surface plasmon mode [38,42,43]:

(5) k=2πλndsinθ+m.2πΛ=Reksp

where $n_{d}$ is the refractive index of the dielectric surrounding the grating, λ is the wavelength of the incident wave, m is the mode index, and Λ is the grating constant. Coupling can be observed as a dip in the reflectivity curve of the grating [31]. By tracking the changes in the position of this dip, the grating can be used for sensing applications. The profile of the grating can be sinusoidal [31] or rectangular [44]. By controlling the geometrical parameters of the grating, the plasmonic resonance can be tuned [44].

Another configuration for generating a propagating surface plasmonic wave utilizes a dielectric waveguide that is covered by a thin metallic layer (Figure 1D). The operation of this configuration is similar to the Kretschmann configuration. Phase matching is satisfied between the propagating mode in the waveguide and the surface plasmon mode of the metal by adding a guiding layer in between. Surface plasmon waves can be obtained on the external surface of the thin metallic layer [45] by coupling to the evanescent modes of the waveguide [46,47]. A recent approach using this configuration employed optical fiber as the waveguide, where the thickness can be adjusted to tune the plasmonic resonance from the visible to the infrared range [48]. This design offers several advantages such as miniaturized footprint and flexibility. In addition, the use of flexible materials such as polymers provides lower cost and lighter weight [49]. This configuration has, however, degraded performance compared to the Kretschmann configuration [50].

### 2.2. Localized SPR

The LSPR is supported by metallic nanoparticles (NPs) that have the ability to absorb energy from the incident radiation [9,51]. When this absorption is maximum, a peak in the absorption curve takes place, forming a resonant behavior. By tuning the size and shape of plasmonic NPs, plasmonic resonance can be shifted towards spectral regions where metallic losses are small [52]. Multiple resonances can be obtained by imposing asymmetry in the shape of NPs [9].

**Figure 2:**
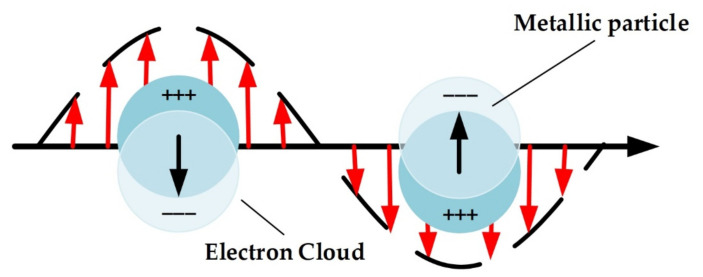
Schematic for localized surface plasmon.

Figure 2 shows the localized surface plasmon. When NPs are illuminated by an incident EM radiation, charge separation of the NPs takes place, leading to the formation of polarization vectors. Due to the oscillatory nature of the incident wave, the generated polarization oscillates in the same way [53]. The collective oscillation of the electrons indicates coupling of energy from the incident radiation and so absorption takes place. A measure of the interaction between incident radiation and NPs is the extinction cross-section, which is defined as the ratio between the sum of the energies absorbed and scattered by the plasmonic NPs to the energy of the incident wave [9]. For a spherical nanoparticle, the extinction cross section can be written as [38]:

(6) $$\sigma_{\mathrm{ext}}=\frac{24\pi^{2}}{\lambda }\varepsilon_{d}{}^{1.5}a^{3}.\frac{\varepsilon_{\mathrm{mi}}}{{\left(\varepsilon_{\mathrm{mr}}+2\varepsilon_{d}\right)}^{2}+\varepsilon_{\mathrm{mi}}{}^{2}}$$

where a is the radius of the nanoparticle. This equation shows that the interaction between the plasmonic nanoparticle and the incident radiation is affected by several parameters, such as the permittivity of the surrounding medium ($\varepsilon_{d}$), the values of the real and imaginary parts of the permittivity ($\varepsilon_{\mathrm{mr}}$ and $\varepsilon_{\mathrm{mi}}$), and the size of the nanoparticle.

The polarization is maximized when the denominator approaches infinity. This occurs when the real part of the metal permittivity is negative with a modulus equal to $2\varepsilon_{d}$ [38], and the imaginary part of the metal permittivity approaches zero.

The plasmon resonance frequency can be controlled by changing the shape and aspect ratio of the NPs [29,54]. In this case, the factor $\left(2\varepsilon_{d}\right)$ in Equation (6) is replaced by ${\gamma \varepsilon }_{d}$, where γ is a factor that is dependent on the particle shape [55,56] Scattering by homogeneous spherical NPs can be analyzed using Mie theory [56]. To extend the analysis to non-spherical particles, the discrete dipole approximation method can be employed [57,58] Extinction of a nanoparticle is the sum of its absorption and scattering effects. For small-shaped NPs, the absorption dominates the extinction, and as the size increases, the scattering effects become more dominant [9,29]

In the case of LSPR, smaller footprint and multiplexed plasmonic spots can be achieved, and it does not need bulk setup for coupling with the incident waves [56,59]. In addition, it is less sensitive to analytes away from the surface, so it is more robust against interference [55]. On the other hand, biosensors based on propagating modes such as Kretschmann configuration involve thin metal films that couple with evanescent modes of a close dielectric. These structures provide robust biosensors with high sensitivity, but large components thwart miniaturization of the biosensors.

## 3. Plasmonic Materials

For effective biosensing of analytes, precise control of plasmonic resonance, including wavelength and angle, is required. In addition, structures that support the formation of hot spots where light is confined are needed for analyte manipulation. These requirements can be met by using nano-patterned materials with a negative real part permittivity over the frequency band of interest. Key parameters that affect the choice of a plasmonic material are the plasmonic losses, chemical stability, resonating frequency range, and integration compatibility. Table 1 shows the most widely used plasmonic materials with their typical properties and operating wavelengths. In this section, we discuss the properties, limitations, and applications of different types of plasmonic materials. We also highlight the polymer substrates used in fabricating flexible plasmonic structures.

### 3.1. Metals

Metals are the first choice of materials for plasmonic applications because they naturally possess negative real permittivity in the visible and near-infrared ranges and have good electrical conductivity [30]. Among metals, silver is the most widely used plasmonic material due to its low losses, strong resonance, and long propagation length [12,63]. The study in [64] reviews the synthesis of different shapes of silver NPs such as nanocubes, nanospheres, nanoprisms, and pyramids. It shows that by tuning the size and shape of the NPs, plasmonic resonance can be obtained over the whole visible spectrum.

**Figure 3:**
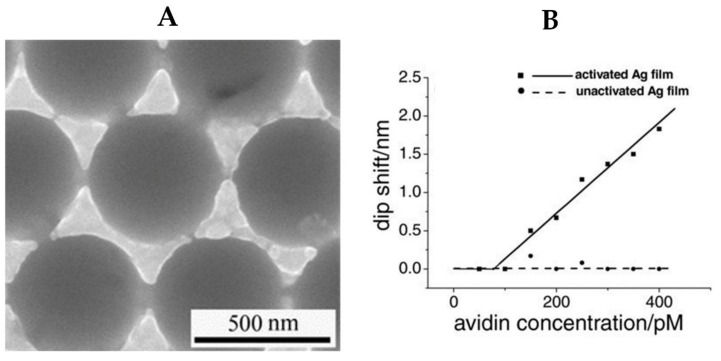
(**A**) Scanning electron microscopy (SEM) image of a porous silver thin film for sensing. (**B**) Activated silver film as a biosensor for detection of avidin [78].

Silver NPs are sensitive to a surrounding analyte and thus can be used for biosensing. This sensitivity can be obtained by shining the silver NPs with EM radiation within the resonant range and measuring the absorption spectrum. To achieve analyte-selective sensing, silver NPs are functionalized with specific chemical groups such as thiols to enhance the binding of the NPs to a specific analyte, then the shifts in their LSPR during exposure to different concentrations of the analyte are monitored. For example, the work in [78] employed a 500 nm porous silver film for the detection of avidin molecules (Figure 3A). The silver film was modified using covalent binding with biotin, which is known for its high affinity to bind to avidin molecules. This work emphasized the importance of functionalization of the silver thin film to activate the sensing, as the non-functionalized film does not show any sensitivity to the change of concentration of avidin molecules (Figure 3B). Another example is the work in [79], which reports using silver nanoprisms for colorimetric glucose sensing. Glucose oxidase is first added to a homogenous solution of silver nanoprisms. The oxidation process produces hydrogen peroxide, which etches the silver nanoprisms to nanodisc shapes, thus shifting the plasmonic resonance from the blue region to the mauve region.

Although silver shows the strongest plasmonic resonance in the visible range, it suffers from chemical instability and toxicity, leading to dull plasmonic resonance and reduced biocompatibility [79]. To solve this problem, a green method was employed in [80] using a natural polymer to fabricate biocompatible silver NPs of sizes between 2 nm and 30 nm. Another method is to passivate the silver film surface using atomic layer deposition [81]. Another challenge for silver is its incompatibility with silicon manufacturing technologies [12], which renders it unsuitable for integrated CMOS devices.

**Figure 4:**
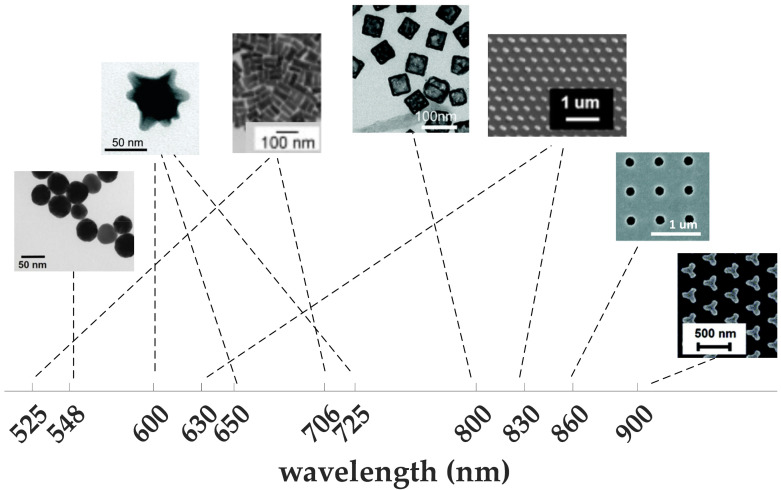
Versatility of gold nanoparticles; (from left to right) nanospheres [60], nanostars [88], nanorods [89], nanocages [92], nanodiscs [90], nanoholes [93], and iso-Y [97], to support plasmonic resonance all over the visible range. Nanostars and nanodiscs can support multiple resonances.

Gold is the best material that can address the instability challenges of silver. It is characterized by its high biocompatibility, high chemical stability, and ease of surface functionalization [82,83]. All these factors make it favorable for biomedical applications such as bio-detection and drug delivery [84,85]. The precise control of the synthesis process allows for realization of different shapes and sizes of gold NPs [85,86,87] (see Figure 4), with the ability to support multiple LSPR using anisotropic structures such as nanostars [88], nanorods [89], and nanodiscs [90]. Gold nanorods are characterized by enhanced absorption compared to other shapes, such as nanospheres and nanoshells [91]. To detect analytes with very low concentration, gold nanocages proved to achieve a limit of detection (LoD) that is an order of magnitude lower than that of nanorods [92]. Gold nanoholes are another popular structure that allows for collinear excitation and detection of SPR in transmission mode [93,94]. The structure can support LSPR in the form of strong field confinement at the edges of the holes [95]. In [96], an array of gold nanoholes is used for sensing of a protein layer of 3 nm thickness. The protein layers can be identified from observing the difference in the diffraction pattern of the array before and after functionalization with the protein layer, which is a label-free technique that provides miniaturization and easier integration. With appropriate functionalization, gold nanoparticles can even provide high specificity to particular analytes [97].

Challenges that are encountered with using gold as a plasmonic material include its high cost and poor adhesion to silica substrates [69]. To improve the adhesion, layers of materials such as chromium and titanium are typically used [98,99]. However, inclusion of these layers directly affects the strength and location of the plasmonic resonance [100]. Moreover, it was shown that smaller roughness and bigger grainsize play an important role in plasmonic sensor applications [69]. Another challenge is the incompatibility of gold with standard silicon fabrication techniques [12]. This limits the applicability of gold nanostructures in integrated CMOS systems, as in the case of silver.

Low-cost fabrication and process compatibility with standard silicon technologies are two important aspects in the selection of plasmonic materials. Aluminum (Al) possesses both of these criteria; thus, it is a reasonable alternative to gold and silver [12,101]. It exhibits plasmonic resonance in the ultraviolet range, at which many organic materials have strong absorption properties [102]. The authors in [101] showed that aluminum nanodisks could be used to obtain plasmonic resonance between 300 nm and 550 nm by altering the diameter of the nanodisks between 70 nm and 180 nm. Resonance can even be tuned down to 270 nm by decreasing the size of Al NPs down to 50 nm [103]. The nanoholes configuration is best suitable for integration of Al for LSPR sensing in integrated chips [104]. Specimens of Al nanoholes deposited on glass and polycarbonate substrates showed spectral shifts when they are immersed in liquids with different refractive indices [104,105], which proved the potential of the structure for biosensing.

One of the main challenges of Al is its high chemical instability. Al is highly reactive with the surrounding atmosphere, leading to the formation of an oxide layer, which degrades the strength of the plasmonic resonance [101]. The aluminum surface can be coated to reduce the oxidation effect. For example, a polydopamine layer was used in [106] to protect an Al array of nanodots from corrosion. Additionally, an aluminum surface can be passivated via plasma treatment, resulting in oxide layers that are highly resistant to oxidizing agents [105].

Copper is a low-cost and high conductivity plasmonic material, with excellent compatibility with CMOS technologies [12], making it highly promising for integrated nanophotonic applications. Copper can support plasmonic resonances in the near-infrared range that can outperform that of gold [69,107]. The plasmonic resonance of copper NPs was exploited in several biosensing applications such as pathogen detection and glucose sensing [65]. The study in [66] showed that copper can be used as a biosensing platform by integrating it as a coating to a photonic crystal fiber.

Similar to Al, copper is highly prone to surface oxidation [108]. As mentioned, copper oxides deteriorate the plasmonic behavior of copper. Several strategies can be followed to overcome this problem, such as oxide removal, slowing down the reaction using reducing agents, and copper surface passivation [109].

### 3.2. Nonmetals

Materials with high thermal stability and hardness are required for applications in harsh environments, such as high temperature. Titanium nitride (TiN) is a plasmonic material characterized by high stability, hardness, and high melting point [72], which makes TiN particularly suitable for high-temperature applications such as photothermal therapy [110], and as electrodes for bio-electrochemical sensing [111]. In addition, the fabrication techniques of TiN are compatible with CMOS fabrication techniques, which makes it promising for chip integration [112,113,114]. TiN is a stoichiometric material whose properties depend on the fabrication parameters, such as deposition temperature and metal/nitrogen ratio [115]. In addition, the substrate and sputtering method selection are crucial to control the resulting metallic properties [112,116].

**Figure 5:**
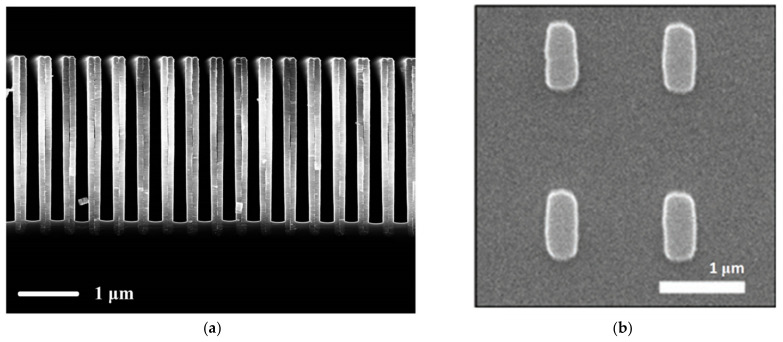
(**a**) Array of TiN trenches used for refractive index sensing in the visible range (Reprinted with permission from [122] © The Optical Society (**b**) Array of plasmonic InAsSb antennas on GaSb substrate for SERS [127].

TiN exhibits plasmonic resonance in the visible and NIR ranges such as gold [117], and thus can be used as a low-cost alternative for gold to achieve acceptable sensitivity at the expense of a higher LoD [118]. For example, an on-chip waveguide was demonstrated in [73] having a figure of merit better than gold due to the increased propagation length. A recent configuration of interest shows the use of TiN as a coating to a photonic crystal fiber (PCF) for refractive index sensing [119,120]. The sensing mechanism depends on the overlap between the plasmonic modes of the TiN coating and those of the supporting fiber. The studies did not include any experimental trials due to the complicated fabrication procedure required, that involves precise removal of a section of the PCF and extra polishing [120,121], which would be an interesting future research area. A more fabrication-feasible grating structure was studied in [122] and the experimental results showed that it can be used for sensing the refractive index of different liquids, such as ethanol and isopropanol (Figure 5a). The functionalization of TiN thin film with biotin was demonstrated in [123], and was used for streptavidin sensing. Nevertheless, employing TiN in biomedical applications requires complex fabrication techniques to maintain its biocompatibility and prevent residual contamination [124]. Another challenge in employing TiN for plasmonic applications is that the plasmonic modes have very short propagation length compared to that of gold and silver [125]. In addition, studying the plasmonic properties of chemically synthesized TiN powder is needed for large-scale fabrication [126].

Both metals and 2D materials (discussed later in this subsection) offer good plasmonic responses but most of these materials are not compatible with existing mature CMOS integration methods. This eliminates the ability for large-scale integration of plasmonic materials and devices with electronic devices. Doped semiconductors, on the other hand, are widely used in integrated circuits. For non-doped semiconductors, the charge carriers’ density is very low compared to that in metals. To have an effective charge oscillation, doping should be performed [128,129]. The real part of the permittivity of these materials can be tuned based on the doping level thus enabling them to behave as metals in the near-infrared range [128].

An important area that can benefit from the advances of plasmonic doped semiconductors is surface-enhanced Raman spectroscopy (SERS), which is a technique that spectrally analyzes the chemical and biological properties of analytes via deep interactions at the plasmonic surface level [130]. In [127], the authors demonstrate the use of an InAsSb antenna array deposited on gallium antimonide (GaSb) for SERS of vanillin (Figure 5b). The field enhancement concentrated around the corners of the nanoantennas is used as the main property for sensing. The main advantage of the structure is the utilization of the overlap between the longitudinal and transverse resonances for providing stronger enhancement factors in the mid-IR range. This cannot be achieved with gold nanoantennas operating in the same range because of the high aspect ratio of the gold elements, thus having no overlap between the longitudinal and transverse resonant modes.

**Figure 6:**
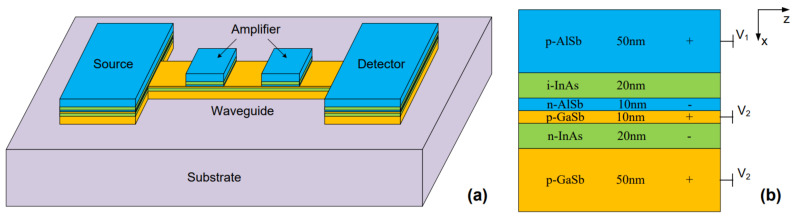
(**a**) Three-dimensional view and (**b**) side-view of an all-semiconductor structure that shows the feasibility of monolithically growing all components of plasmonic system (source, guide, and detector) on a chip. Three types of doping (n-type, p-type, and i-type) are proposed to provide carriers for plasmonic excitation (Reprinted with permission from [131] © The Optical Society).

Figure 6 shows an example of a monolithically integrated all-semiconductor system that supports a propagating SPR at the interface between n-doped indium arsenide (InAs) and p-doped GaSb layers [131]. This material system exhibits good plasmonic behavior in the mid-infrared range between 8 μm and 12 μm. Although the structure illustrates a good example of integrating different components of a plasmonic operation, material selection is not optimal for sufficient plasmonic charge accumulation [71]. The authors in [44] showed that the size of a plasmonic grating of InAsSb is four times smaller in area than a plasmonic grating of gold resonating at the same wavelength, which provides a path to device miniaturization. Si-doped indium phosphide is another interesting semiconductor that shows good propagation length and low loss in the range between 10 μm and 30 μm [132].

The main challenge in using doped semiconductors as plasmonic materials is the issues with solid solubility resulting from the doping process [30,127]. Reaching sufficient carrier density via doping is feasible in the THz range, but it is much more challenging in the mid-IR range as it requires very high values of voltage, and the homogeneity of the resulting doped structure is not guaranteed [71].

Two-dimensional nanomaterials are crystalline layered materials, characterized by direct bandgaps and ultrahigh conductivity, and they possess excellent electrochemical properties, including sensitivity to optical excitation, due to their large surface area, high carrier density, and mobility [133]. Their ultrathin thickness provides strong light–matter interaction, which makes them good candidates for building biosensors [134,135]. Unlike metals, their carrier concentration can be tuned to adjust the frequency range of their plasmonic behavior [135]. In their intrinsic form, 2D materials exhibit plasmonic resonance in the mid to far IR range with limited applications. By doping these materials, their plasmonic resonance can be shifted to the visible and near IR ranges [17,135]. Table 2 shows the main properties of some important 2D materials recently investigated for their plasmonic properties.

**Figure 7:**
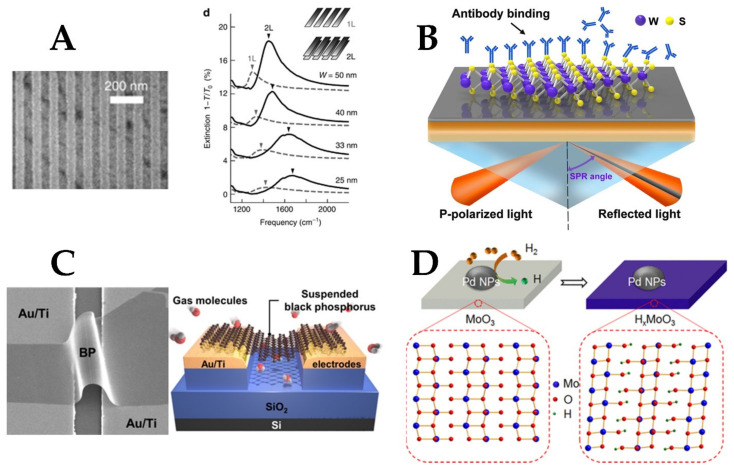
(**A**) Controlling the plasmonic resonance of graphene by changing the number of monolayers [145]. (**B**) Using WS2 as a binding surface for plasmonic sensing using Kretschmann configuration [147]. (**C**) Black pShosphorus deposition as a suspended layer between metal electrodes [138]. (**D**) N-type doping of molybdenum trioxide using hydrogen atoms. Chemisorbed hydrogen molecules dissociate at metal sites, eventually diffusing into the bulk, leading to the reduction of the semiconductor [148].

Graphene is a promising 2D material for plasmonics due to its high electron mobility and high mechanical flexibility [99]. Graphene proved to accommodate surface plasmons that can be observed from the far-infrared to mid-infrared range [16]. The conductivity of graphene can also be tuned using electrostatic gating, making it possible to achieve plasmonic properties in the mid-IR range [144]. Moreover, the plasmonic resonance strength can be adjusted by changing the number of graphene monolayers (see Figure 7A) [145]. The plasmonic absorption in graphene structures such as ribbons array can be enhanced in the mid-IR region by coupling them with metal gratings [16,75]. In [146], graphene layers were placed on top of a gold sensing film, resulting in improved sensitivity of the device due to the strong field confinement induced by graphene. In addition, the direct electrical contact between graphene and gold layers formed an interface supporting surface plasmon polaritons.

Biosensors employing graphene layers were demonstrated in [149,150,151]. A linear relationship was established between the number of graphene layers and the enhancement in sensitivity [149]. However, as the number of added layers increases, the plasmonic resonance becomes broader, leading to a decline in the sensing performance [151]. Graphene layers were used in [150] on top of gold and silver layers to enhance the sensitivity, and form a passivation layer that hinders the oxidation of the sensing surface. One of the challenges that are encountered when using graphene as a plasmonic material is the difficulty of its pattering with reduced edge effects. These challenges can be overcome by investigating chemical synthesis methods such as block co-polymer lithography [152,153].

Transition metal dichalcogenides (TMDs) are another group of 2D materials that are characterized by having a very thin structure and a tunable bandgap [134]. The basic monolayer is formed from a layer of transition metal sandwiched between two layers of chalcogenide material (see Figure 7B) [148]. An interesting property of TMDs is that the indirect bandgap in bulk becomes a direct bandgap in the monolayer form, which allows the 2D material to directly absorb or emit photons if the external energy is larger than the bandgap [147]. An example of TMDs is molybdenum disulfide (MoS_2_), which is characterized by its low toxicity [134]. In addition, the very thin nature of MoS_2_ layers makes it very sensitive to binding analytes that directly change its thickness and so can be highly detected [134].

Despite the fact that 2D TMD has strong light–matter interactions, they have low optical cross-section value which results in low absorption, and accordingly decreases its efficiency in applications such as energy conversion, light harvesting and sensing. Two-dimensional TMD hybridization with plasmonic metal is a promising technique to improve optical absorbance, and therefore sensitivity, in various applications. In [17], the MoS2 monolayer was functionalized with gold nanoparticles, leading to an improvement in light absorption by 35%. Another tunable property of TMDs is photoluminescence (PL), by hybridizing monolayer of TMD with shape-controlled plasmonic particle. One example is Ag nanocube/MoS2 monolayer hybrid structure, which showed ~2 times enchased PL compared to bare MoS2 monolayer [154].

Layered black phosphorus (BP) is another interesting 2D material with a unique anisotropic structure, high carrier mobility, and tunable bandgap [155]. It overcomes the challenge of zero bandgap in graphene while maintaining higher carrier mobility than TMDs [138]. In [138], the authors experimentally demonstrated the gas sensing using a BP layer suspended between the electrodes to increase the adsorption sites and avoid scattering effects (Figure 7C). BP can also be hybridized with noble metals, such as gold and silver to enhance the sensitivity, which can also be controlled by changing the number of BP layers [154,155]. However, BP is very unstable in ambient conditions and there is a lack of large-scale fabrication methods [156]. One way to isolate its surface is by using a fully oxidized BP layer as a capping layer [157], a technique similar to that used for aluminum passivation.

One way of inducing charge transfer in 2D materials is via doping. An interesting example is the n-doping of 2D molybdenum trioxide (MoO_3_) using hydrogen (see Figure 7D). Through an intercalation process, H+ atoms are introduced to fill the gaps within MoO_3_ nanoflakes, oxygen vacancies are induced, leading to enhanced absorption in the NIR [135,148] and visible ranges [148]. MoO_3_ was investigated in [135] for sensing of albumin and the results showed a detection limit down to a concentration of 1 picogram per milliliters. The design integrates MoO_3_ nanoflakes to a micro-optical fiber to benefit from the fiber flexibility and miniaturized size.

### 3.3. Substrates

A substrate is a key component for holding plasmonic structure and providing wearable application-oriented requirements such as mechanical flexibility and electrical isolation. Silicon and glass are commonly used substrates that are rigid and cannot be meandered [158]. Hence, these substrates may not be suitable for future biosensors that require a flexible and biocompatible substrate [159]. Polymers have emerged as a main choice for the substrates because of their flexibility, biocompatibility, and low cost. In addition, polymer substrates can eliminate the need for using an adhesion layer between the plasmonic structure and the substrate [160]. Several types of polymers have been utilized in plasmonic applications. In this section, we cover the properties and applications of some major polymers employed for plasmonic devices. Table 3 shows the main properties of the polymer substrates studied in this section. The reader interested in more details about polymer integration techniques may refer to the review study in [161].

**Figure 8:**
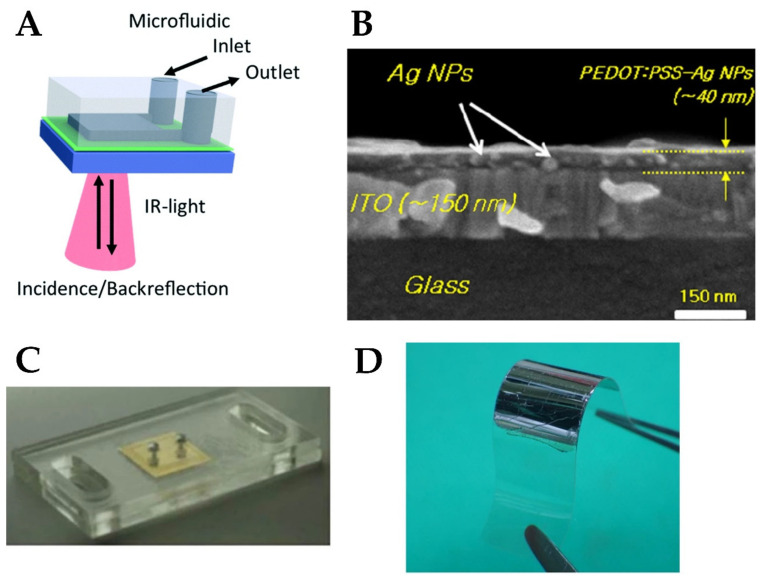
(**A**) SPR sensor using PDMS microfluidic channel [176]. (**B**) Integration of PEDOT:PSS with silver NPs in organic solar cell [177]. (**C**) A PMMA microfluidic chip with plasmonic gold coating for biosensing [41]. (**D**) Integration of silver nano-array to a flexible PET substrate [178].

Low-cost, flexible substrate is required for the implementation of ubiquitous wearable biosensing devices. Polydimethylsiloxane (PDMS) is one of the low-cost, flexible polymers possessing excellent elastic properties. It is non-toxic, has good thermal and oxidation stability, and is easily fabricated. More importantly, it is optically transparent over a broad range of frequencies [174]. It is considered the optimal choice for the fabrication of microfluidic channels which are an essential component in plasmonic biosensors for the efficient binding of analytes to the sensing device [175]. For example, the authors in [176] employed PDMS microfluidic for measuring the amount of alcohol in a steady flow of liquid by correlating the alcohol content with shifts in the plasmonic resonance of a silicon-doped InAs film (schematic shown in Figure 8A). Due to its excellent elasticity, a hybrid structure consisting of Au core/Ag shell nanorods onto PDMS showed tunable plasmonic resonance by applying and releasing external compression force on the substrate [162].

One challenge of the PDMS substrate is its intrinsic hydrophobicity, which hinders the cell adhesion process [179], and also may lead to non-specific binding issues where small molecules secreted by the analyte cells are unintentionally adsorbed [180]. Another challenge is that PDMS is incompatible with organic solvents, where these solvents would diffuse through PDMS and change its properties [163].

Conductive polymers play an important role in the plasmonic operation as sensing electrodes and connectors. Polyethylene dioxythiophene: polystyrene sulfonate (PEDOT:PSS) mixture is a conductive polymer with high transparency in the visible range, which is further characterized by high flexibility, thermal stability, and ease of fabrication [181]. Its low dielectric permittivity makes it easier to attain surface plasmon polariton coupling with metals such as silver [143]. In addition, the organic nature and softness of PEDOT makes it more suitable for attaching biomolecules [182]. These interesting properties have qualified PEDOT:PSS to be used in different sensing applications such as organic connectors for biosensors [183], glucose sensing [184], and organic electrochemical transistors [185]. Using PEDOT:PSS in organic solar cells (Figure 8B) is another interesting research area for powering future flexible biomedical devices [177].

PEDOT:PSS is a promising material for the replacement of indium tin oxide (ITO) as a conductive electrode, but the challenge is in its low conductivity compared to ITO. Another challenge is its acidity which leads to the degradation of the active layer [186]. Several techniques have been used to improve the conductivity, such as treatment with dopants, direct dilution, and acid treatment [169].

Thermoplastic substrates are a good candidate to enhance the integration of plasmonic devices; since they can be melted and cooled down to solidify over many cycles without alteration of their properties, which provides more control over the shape and pattern [187]. PMMA is a thermoplastic polymer that possesses interesting properties, such as high transparency, high flexibility, and biocompatibility [188]. The high optical transparency of PMMA makes it suitable for fabricating nanocomposites with embedded plasmonic nanostructures that can be used for efficient sensing, such as silver [167] and gold composites [41,189] (Figure 8C).

The study in [189] highlights the role of the post-annealing step of the gold-PMMA composite above the glass-transition transition temperature of PMMA in increasing the sensitivity of the structure due to the transition of the polymer from glass state to rubbery state, which would lead to increasing the mobility of polymer chains.

One main challenge of PMMA is its poor gas permeability [163]. This can hinder enzymatic reactions needed for biosensing. For example, enzymatic sensing for glucose monitoring requires membranes with high oxygen permeability; in this case, PMMA would not be a good choice [190].

A low-cost, flexible, and stable substrate would be an ideal substrate for plasmonic sensing if the substrate surface can be functionalized to bind with analytes. Polyethylene terephthalate (PET) is a flexible, lightweight, and low-cost polymer [191] with high thermal stability and conductivity [192]. In addition, it has good mechanical properties and solvent resistance, and so has found applications in biological implants, such as artificial heart valves and blood vessels [193].

PET was used in [194] as a flexible substrate for piezoelectric energy harvesters with robust performance when subjected to many bending cycles. It was used as a flexible substrate for an array of silver NPs with bending capability up to a curvature of 1 mm (Figure 8D) [178]. The use of PET as a substrate for antigen–antibody reaction biosensing was demonstrated in [191,195].

However, PET in its original state is not suitable for NPs deposition as it is normally hydrophobic and has weak adhesion [196]. To overcome this challenge, plasma treatment can be used to increase the wettability of the PET surface [197,198].

Polycarbonate (PC) is another example of thermoplastic substrates with high optical clarity [199], and low-cost [200]. It is the main material used in the manufacturing of compact disks (CDs) [201]. PC-based CDs show good integrity with microfluidics due to the ease of control of fluids using centrifugal force induced by compact disc spinning. PC substrate outperforms glass for arrays of gold nanoslits due to better surface smoothness of the gold structure on PC [202]. PC substrate fabricated using anodic alumina templates was demonstrated for SERS [187,200].

One of the challenges in the functionalization of PC is its hydrophobicity [203]. This can be solved by UV treatment, which renders it suitable for building biosensing devices [201]. Another challenge is the lack of mechanical hardness as the surface is prone to degradation upon subjection to UV radiation [204].

## 4. Advantages of Plasmonic Biosensors

Plasmonic-based biosensors continue to gain attention from researchers and the public due to their distinct advantages for healthcare applications. The fundamental difference between traditional and plasmonic-based biosensors is their sensing method. Traditional biosensors implement biochemical and cell assays based on label-based detection, using labels such as enzymes or fluorophores. In contrast to traditional biosensors, plasmonic biosensors use label-free analytical technology, which serves as their main advantage. The most common plasmonic metal is gold; it has extraordinary properties of biocompatibility [205]. In labelled assays, the analyte being measured is captured between a capture and detector agent. The capture agent is commonly immobilized on the surface of a solid, such as a gold sensor chip or an electrode, whereas the detector agent is bound to the label that is used to measure the presence of the analyte. The overall structure of a labelled biosensor is complex and increases the cost of the sensor, as expensive labeling reagents and protocols may need to be used. In label-free biosensors, biochemical reactions on the surface of the plasmonic source are used to detect the presence of the analyte. Label-free biosensors only require one recognition element for a given analyte. Furthermore, label-free biosensors can measure biochemical interactions in real-time to provide continuous monitoring of data.

Plasmonic biosensors, which utilize SPR- and LSPR-based mechanisms, are low-cost devices that can be easily miniaturized compared to other techniques that require more time and skill, such as polymerase chain reaction (PCR) and enzyme-linked assay methods. LSPR biosensors have a much simpler optical configuration, which does not require a prism to couple the light [55]. In a LSPR device, the plasmons excited by the incident light oscillate to the nanoparticle itself, rather than across the metal–dielectric interface as in SPR.

Consequently, the electromagnetic field decay length of localized surface plasmons is much shorter compared to the surface plasmon radiation [206]. The plasmonic biosensors use an optical method to measure changes in the refractive index of a film induced by bio-molecular interactions at the surface. In the sensor, a light source shines incident light with properties specific to the system onto the sensor film. This light is then reflected and captured by the detection system, which then detects the intensity of the reflected light as well as the resonance absorption peak. Biomolecular interactions on the surface of the sensor are translated into changes in the refractive index of the film, which impact various properties such as the resonance wavelength, resonance angle, and the intensity of the reflected light. Through analyzing these properties, biomolecular interactions and the presence of specific molecules can be detected by the sensor. There are several mechanisms and detection methods where a plasmonic sensor can operate. The more commonly used detection method uses monochromatic or polychromatic incident light. The change of the incidence angle results in a variation of the reflected light intensity. The incident angle is referred to as the angle of resonance when the intensity of the reflected light reaches its lowest value. When polychromatic incident light is used, the wavelength of the light is changed while the incident angle is kept constant. These changes lead to changes in reflectivity, which are then analyzed to find the resonant wavelength. Other less common methods function through fixing both the angle and wavelength of the incident light. This can be used to detect changes in the refractive index by analyzing variations in the reflected light intensity or to measure the phase difference between the incident and reflected light [36].

The performance of a plasmonic sensor is essential to making it suitable for biosensing applications. The performance of sensors is measured through their sensitivity and their figure-of-merit (FOM), which is the ratio between the sensitivity and the full width at half maximum (FWHM) of the resonance wavelength [59,207]. Although plasmonic sensor instrumentation has made significant advancements over the last decade, efforts are continuously being made to improve its performance.

The FOM of LSPR biosensors are low compared to SPR biosensors due to the radiative damping in the LSPR modes, as this leads to an increase in the resonance peak width. Due to this low FOM, the performance of the LSPR sensor is significantly reduced. Researchers have shown that the nanostructures’ size, shape, and material are essential in enhancing the performance of the LSPR sensor; however, tuning these properties of the NPs has yet to increase the sensor’s performance to a level comparable to SPR sensors [208]. To further optimize the performance of the LSPR sensor, surface plasmon hybridization can be used to increase the sensitivity of the biosensor.

**Figure 9:**
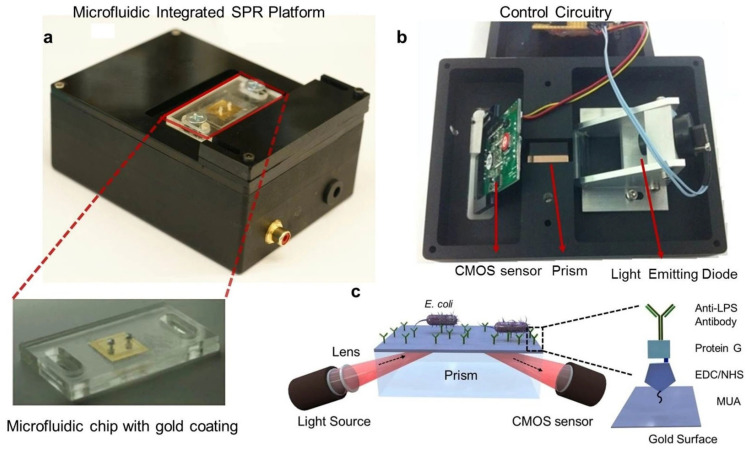
A portable plasmonic biosensing system that includes the light source, sensor chip, and detector, in addition to a microfluidic channel for analyte manipulation. (**a**) The portable integrated package (**b**) The Kretschmann configuration implemented using LED source for excitation, a prism for plasmonic coupling, and a CMOS sensor for reflected wave detection. (**c**) An example of using the portable platform for biosensing of E. coli bacteria. Surface modification was performed to improve binding between the analyte and the sensing surface [41].

Figure 9 shows an example of a portable integrated plasmonic biosensing system [41]. The device employs SPR excitation using prism coupling, which increases the size of the package. In addition, space is required to maintain total reflection through the prism. An alternative route to miniaturize such a system is by using waveguide coupling between the source and detector. A III-V material can be used within a quantum well to generate light for plasmonic excitation [209]. The plasmonic sensor would be placed over the waveguide to couple with the guided modes when the wave matching condition is achieved. A photodetector can then be used to monitor the changes in the wave induced by SPR. A 2D material such as graphene can be used to enhance photodetection efficiency.

## 5. Technological and Economic Challenges

Due to the bulky setup needed for SPR-based devices, LSPR technology is more accessible in integrated and portable structures. LSPR sensors, however, still suffer from some technical difficulties. We summarize these challenges in three main categories:

### 5.1. Fabrication-Related Challenges

Reproducible fabrication of high-performance plasmonic devices over a large area in a low-cost, high-throughput manner is still a big challenge [210]. Serial top-down approaches such as electron-beam lithography and focused-ion beams are not suitable for mass production due to fabrication complexity, materials wastage, and high cost. Other fabrication techniques such as nanoimprint lithography and nanostencil lithography will play an important role in decreasing the fabrication cost [77]. However, the deterministic formation of sensing hotspots using bottom-up techniques and active delivery of analytes to these hotspots remains an open challenge [211]. In addition, the repeatability of these synthesis methods cannot be guaranteed, leading to differences in plasmonic signals which would affect the sensing performance [212]. With LSPR gaining more interest for portable applications, more efforts should be taken to improve the adhesion between plasmonic nanoparticles and flexible substrates [213].

### 5.2. Operation-Related Challenges

Non-specific binding is another problem related to the specificity of the LSPR sensor [19]. This happens when different analytes couple to the surface of the plasmonic biosensor, leading to erroneous measurements. More research should be conducted on new functionalization methods to enhance the specific binding ability of the sensor. For more integrated setups, the miniaturization of light sources is still a challenge [77]. As most excitations depend on using an external source to shine the sensor surface, the device becomes bulky and not suitable for portable applications [214]. Cavity-induced laser sources are promising technology for integrated light generation [209], but coupling efficiencies are still not adequate. As the portable biosensors detect trace amounts of analytes, there is a need to efficiently clean the microfluidic channel between different sensing cycles [215], which would also dampen the effect of non-specific binding.

### 5.3. Performance-Related Challenges

Sensitivity values of LSPR are still low compared to those of SPR setups, which hinders the wide use of LSPR for portable applications such as point-of-care. Since LSPR structures rely on solution-based methods of fabrication, several factors affect the resulting device such as ambient noise, detector resolution, and analyte amount [216]. Point-of-care applications require sensing of analytes at very low concentration, and under these external effects the signal-to-noise ratio becomes very low [19].

## 6. Conclusions and Future Perspectives

Plasmonic materials constitute a breakthrough in the advancement of biosensors. This could not be possible without the great advances in fabrication and synthesis techniques that can produce nanoscale structures with precise and reproducible features. In this review, we have summarized the recent advances and research challenges of using plasmonic materials for the integration of biosensor systems. We first went through the basic principles of plasmonics in its propagating (SPR) and localized (LSPR) modes. We identified the main material properties affecting the strength of the obtained plasmonic effect such as the size, shape, permittivity, choice of surrounding dielectric, aspect ratio, fabrication conditions, and coupling between different components in a plasmonic system.

The push for integrated plasmonic systems still faces several challenges, such as the incompatibility of metal plasmonic materials with CMOS integration technologies, the high loss, and the lack of reproducibility for nanoplasmonic devices. New materials are needed to encounter these challenges. Titanium nitride and 2D materials such as graphene, TMD hybrid structures are promising for integrated plasmonic biosensors due to their high chemical stability, tunability, mechanical flexibility, and ease of processing at low-cost.

Future biosensing devices will encompass integration of metallic NPs with 2D semiconductor sheets. This will combine the efficient plasmonic properties of metals with the tunability and ultra-thinness of 2D layers. In addition, 2D sheets would provide shielding for stabilizing NPs and prevent their aggregation during fabrication. We proposed a few hybrid structures which showed a significant improvement in absorbance and photoluminescence applications.

Polymer substrates provide an effective platform for flexible and wearable devices. Factors affecting the choice of polymer include surface wettability, surface roughness, thermal stability, and biocompatibility. Future integrated systems include wearable devices that operate under various conditions, such as stretching, bending and twisting. More investigation is needed to design plasmonic components that maintain robust performance under such harsh conditions. In addition, new polymers with better wettability and resistance to corrosion are needed to ameliorate the analyte manipulation and support advanced functionalization agents.

The role of artificial intelligence will increase in future plasmonic biosensors for analyzing complex sensor data [217], reducing signal interference [218], removing of noisy signals [218], and high-throughput screening [219]. We propose the following ideas as future research directions for plasmonic biosensors:

- Enhance the adhesion between plasmonic nanoparticles and flexible substrates; either using new polymer material substrates or by controlling the synthesis process.
- Enhance the coupling efficiency of waveguide coupling for integrated light generation and detection.
- Demonstrate experimental approaches for TiN in photonic crystal fiber biosensors and enhance biocompatibility for wide range of applications.
- Improve bottom-up fabrication technologies for reproducible biosensors, and consequently better large-scale adsorption.
- Address non-specific binding issues in new functionalization methods by employing artificial intelligence and signal processing algorithms for multiplexed signal analysis.
- Explore new configurations for biosensing with unconventional properties such as metasurfaces.
- Investigate self-powering methods for wearable biosensors such as solar energy harvesting, triboelectric nanogenerators, and thermoelectric generators.

## Figures and Tables

**Table 1:** Major metal and non-metal plasmonic materials.

Material	Plasmonic Resonance	Properties	Challenges	Applications	Reference
Gold	Visible-NIR	Chemical stability-Biocompatibility	High cost-Incompatible with CMOS	Photothermal therapy/Imaging/Drug delivery	[60,61,62]
Silver	Visible-NIR	Long propagation length	Incompatible with CMOS-oxidation	Surface-enhanced Raman Spectroscopy	[12,63,64]
Copper	Visible	Low-cost, compatible with CMOS	Oxidation	Catalysis/Photonic Crystal Fiber	[65,66]
Aluminum	UV	Compatible with CMOS	Oxidation-High losses in visible range	Photodetection/Nanoantennas	[67,68,69]
Doped semiconductors	Mid IR	Compatible with CMOS	Carrier mobility–solid solubility of dopant	CMOS-compatible plasmonic waveguides	[12,70,71]
TiN	Visible-NIR	Hardness-High melting point	Weaker resonance than metals at room temp.-Fabrication of powder with controlled properties	On-chip waveguide/fluorescence coupling	[12,72,73]
Graphene	Far IR to mid IR	Strong field confinement-High tunability	Mismatch between plasmons and free-space photons-Edges effect	Integrated light generation/Photothermal therapy	[74,75,76,77]

**Table 2:** Plasmonic 2D Materials.

Material	Operating Regime	Tunability	Monolayer Bandgap (eV)	Carrier Mobility (cm^2^/V^−1^s^−1^)	Applications	Reference
Graphene	MIR-THz	Doping-gating	0	10,000	Photodetection	[12,16]
MoS_2_	UV-Vis	Doping	1.8	200	Optical spectroscopy/Photodetection	[136,137]
Black phosphorus	MIR-THz	Doping-gating	1.5	1000	Gas sensing	[138,139,140]
MoO_3_	Vis-NIR	Redox reactions	3	1000	Photodetection/Catalysis	[141,142,143]

**Table 3:** Polymer substrates for plasmonic materials.

Material	Glass Transition Temperature [°C]	Water Contact Angle [°]	Main Applications	Challenges	Reference
PDMS	−125	122	Microfluidic channels	Low elasticity–incompatibility with organic solvents	[162,163,164,165,166]
PMMA	105	68	Implants and drug delivery systems	Poor gas permeability	[167,168]
PEDOT:PSS	N/A	10.5	Flexible solar cells	Acidity	[169,170,171]
PET	80	70	Biological implants	Poor wettability–weak adhesion	[172,173]

## References

[1] Yu H., Peng Y., Yang Y., Li Z.-Y. (2019). Plasmon-enhanced light–matter interactions and applications. Npj Comput. Mater..

[2] Boriskina S.V., Ghasemi H., Chen G. (2013). Plasmonic materials for energy: From physics to applications. Mater. Today.

[3] Zia R., Schuller J.A., Chandran A., Brongersma M.L. (2006). Plasmonics: The next chip-scale technology. Mater. Today.

[4] Dionne J.A., Sweatlock L.A., Atwater H.A., Polman A. (2006). Plasmon slot waveguides: Towards chip-scale propagation with subwavelength-scale localization. Phys. Rev. B.

[5] Ding S.-Y., Yi J., Li J.-F., Ren B., Wu D.-Y., Panneerselvam R., Tian Z.-Q. (2016). Nanostructure-based plasmon-enhanced Raman spectroscopy for surface analysis of materials. Nat. Rev. Mater..

[6] Jones M.R., Osberg K.D., Macfarlane R.J., Langille M.R., Mirkin C.A. (2011). Templated Techniques for the Synthesis and Assembly of Plasmonic Nanostructures. Chem. Rev..

[7] Gramotnev D.K., Bozhevolnyi S.I. (2010). Plasmonics beyond the diffraction limit. Nat. Photonics.

[8] Maier S.A., Brongersma M.L., Kik P.G., Meltzer S., Requicha A.A.G., Atwater H.A. (2001). Plasmonics—A Route to Nanoscale Optical Devices *Adv*. Mater..

[9] Giannini V., Fernández-Domínguez A.I., Heck S.C., Maier S.A. (2011). Plasmonic Nanoantennas: Fundamentals and Their Use in Controlling the Radiative Properties of Nanoemitters. Chem. Rev..

[10] Haes A.J., Zou S., Schatz G.C., Van Duyne R.P. (2003). A Nanoscale Optical Biosensor: The Long Range Distance Dependence of the Localized Surface Plasmon Resonance of Noble Metal Nanoparticles. J. Phys. Chem. B.

[11] Homola J. (2008). Surface Plasmon Resonance Sensors for Detection of Chemical and Biological Species. Chem. Rev..

[12] Naik G.V., Shalaev V.M., Boltasseva A. (2013). Alternative Plasmonic M: Beyond Gold and Silver. Adv. Mater..

[13] Agrawal A., Cho S.H., Zandi O., Ghosh S., Johns R.W., Milliron D.J. (2018). Localized Surface Plasmon Resonance in Semiconductor Nanocrystals. Chem. Rev..

[14] Chen J., Badioli M., Alonso-González P., Thongrattanasiri S., Huth F., Osmond J., Spasenović M., Centeno A., Pesquera A., Godignon P. (2012). Optical nano-imaging of gate-tunable graphene plasmons. Nature.

[15] Cudazzo P., Gatti M., Rubio A. (2012). Plasmon dispersion in layered transition-metal dichalcogenides. Phys. Rev. B.

[16] Low T., Avouris P. (2014). Graphene Plasmonics for Terahertz to Mid-Infrared Applications. ACS Nano.

[17] Sriram P., Manikandan A., Chuang F.-C., Chueh Y.-L. (2020). Hybridizing Plasmonic Materials with 2D-Transition Metal Dichalcogenides toward Functional Applications. Small.

[18] Han X., Liu K., Sun C. (2019). Plasmonics for Biosensing. Materials.

[19] Mejía-Salazar J.R., Oliveira J.O.N. (2018). Plasmonic Biosensing. Chem. Rev..

[20] Evanoff D.D., Chumanov G. (2005). Synthesis and Optical Properties of Silver Nanoparticles and Arrays. ChemPhysChem.

[21] Cai Z., Li Z., Ravaine S., He M., Song Y., Yin Y., Zheng H., Teng J., Zhang A. (2021). From colloidal particles to photonic crystals: Advances in self-assembly and their emerging applications. Chem. Soc. Rev..

[22] Mejía-Salazar J.R., Rodrigues Cruz K., Materon Vasques E.M. (2020). Microfluidic Point-of-Care Devices: New Trends and Future Prospects for eHealth Diagnostics. Sensors.

[23] Liao Z., Zhang Y., Li Y., Miao Y., Gao S., Lin F., Deng Y., Geng L. (2019). Microfluidic chip coupled with optical biosensors for simultaneous detection of multiple analytes: A review. Biosens. Bioelectron..

[24] Gao W., Emaminejad S., Nyein H.Y.Y., Challa S., Chen K., Peck A., Fahad H.M., Ota H., Shiraki H., Kiriya D. (2016). Fully integrated wearable sensor arrays for multiplexed in situ perspiration analysis. Nature.

[25] Maier S.A., Atwater H.A. (2005). Plasmonics: Localization and guiding of electromagnetic energy in metal/dielectric structures. J. Appl. Phys..

[26] Zayats A.V., Smolyaninov I. (2003). Near-field photonics: Surface plasmon polaritons and localized surface plasmons. J. Opt. A Pure Appl. Opt..

[27] Boltasseva A., Nikolajsen T., Leosson K., Kjaer K., Larsen M., Bozhevolnyi S. (2005). Integrated optical components utilizing long-range surface plasmon polaritons. J. Light Technol..

[28] Mayer K.M., Hafner J.H. (2011). Localized Surface Plasmon Resonance Sensors. Chem. Rev..

[29] Lee K.-S., El-Sayed M.A. (2006). Gold and Silver Nanoparticles in Sensing and Imaging:  Sensitivity of Plasmon Response to Size, Shape, and Metal Composition.

[30] West P., Ishii S., Naik G., Emani N.K., Shalaev V., Boltasseva A. (2010). Searching for better plasmonic materials. Laser Photon-Rev..

[31] Dostálek J., Homola J., Miler M. (2005). Rich information format surface plasmon resonance biosensor based on array of diffraction gratings. Sens. Actuators B Chem..

[32] Byun K.-M. (2010). Development of Nanostructured Plasmonic Substrates for Enhanced Optical Biosensing. J. Opt. Soc. Korea.

[33] Zia R., Selker M.D., Catrysse P.B., Brongersma M.L. (2004). Geometries and materials for subwavelength surface plasmon modes. J. Opt. Soc. Am. A.

[34] Liu L., Han Z., He S. (2005). Novel surface plasmon waveguide for high integration. Opt. Express.

[35] Prabowo B.A., Purwidyantri A., Liu K.-C. (2018). Surface Plasmon Resonance Optical Sensor: A Review on Light Source Technology. Biosensors.

[36] Spackova B., Wrobel P., Bockova M., Homola J. (2016). Optical Biosensors Based on Plasmonic Nanostructures: A Review. Proc. IEEE.

[37] Guo X. (2012). Surface plasmon resonance based biosensor technique: A review. J. Biophotonics.

[38] Wang D.-S., Fan S.-K. (2016). Microfluidic Surface Plasmon Resonance Sensors: From Principles to Point-of-Care Applications. Sensors.

[39] Yuan D., Dong Y., Liu Y., Li T. (2015). Design of a High-Performance Micro Integrated Surface Plasmon Resonance Sensor Based on Silicon-On-Insulator Rib Waveguide Array. Sensors.

[40] Hill R.T. (2014). Plasmonic biosensors. WIREs Nanomed. Nanobiotechnol..

[41] Tokel O., Yildiz U.H., Inci F., Durmus N.G., Ekiz O.O., Turker B., Cetin C., Rao S., Sridhar K., Natarajan N. (2015). Portable Microfluidic Integrated Plasmonic Platform for Pathogen Detection. Sci. Rep..

[42] Piliarik M., Homola J. (2009). Surface plasmon resonance (SPR) sensors: Approaching their limits?. Opt. Express.

[43] Wijaya E., Lenaerts C., Maricot S., Hastanin J., Habraken S., Vilcot J.-P., Boukherroub R., Szunerits S. (2011). Surface plasmon resonance-based biosensors: From the development of different SPR structures to novel surface functionalization strategies. Curr. Opin. Solid State Mater. Sci..

[44] Barho F.B., Gonzalez-Posada F., Milla-Rodrigo M.-J., Bomers M., Cerutti L., Taliercio T. (2016). All-semiconductor plasmonic gratings for biosensing applications in the mid-infrared spectral range. Opt. Express.

[45] Homola J., Yee S.S., Gauglitz G. (1999). Surface plasmon resonance sensors: Review. Sens. Actuators B Chem..

[46] Ahn H., Song H., Choi J.-R., Kim K. (2018). A Localized Surface Plasmon Resonance Sensor Using Double-Metal-Complex Nanostructures and a Review of Recent Approaches. Sensors.

[47] Wang W., Mai Z., Chen Y., Wang J., Li L., Su Q., Li X., Hong X. (2017). A label-free fiber optic SPR biosensor for specific detection of C-reactive protein. Sci. Rep..

[48] Ahn J.H., Seong T.Y., Kim W.M., Lee T.S., Kim I., Lee K.-S. (2012). Fiber-optic waveguide coupled surface plasmon resonance sensor. Opt. Express.

[49] Lopez G.A., Estevez M.-C., Soler M., Lechuga L.M. (2017). Recent advances in nanoplasmonic biosensors: Applications and lab-on-a-chip integration. Nanophotonics.

[50] Ribaut C., Loyez M., Larrieu J.-C., Chevineau S., Lambert P., Remmelink M., Wattiez R., Caucheteur C. (2017). Cancer biomarker sensing using packaged plasmonic optical fiber gratings: Towards in vivo diagnosis. Biosens. Bioelectron..

[51] Fong K.E., Yung L.-Y.L. (2013). Localized surface plasmon resonance: A unique property of plasmonic nanoparticles for nucleic acid detection. Nanoscale.

[52] Gill R., Tian L., Somerville W.R.C., Le Ru E.C., van Amerongen H., Subramaniam V. (2012). Silver Nanoparticle Aggregates as Highly Efficient Plasmonic Antennas for Fluorescence Enhancement. J. Phys. Chem. C.

[53] Cao J., Sun T., Grattan K.T.V. (2014). Gold nanorod-based localized surface plasmon resonance biosensors: A review. Sens. Actuators B Chem..

[54] Khlebtsov N.G., Dykman L.A. (2010). Optical properties and biomedical applications of plasmonic nanoparticles. J. Quant. Spectrosc. Radiat. Transf..

[55] Hammond J.L., Bhalla N., Rafiee S.D., Estrela P. (2014). Localized Surface Plasmon Resonance as a Biosensing Platform for Developing Countries. Biosensors.

[56] Unser S., Bruzas I., He J., Sagle L. (2015). Localized Surface Plasmon Resonance Biosensing: Current Challenges and Approaches. Sensors.

[57] Miller M.M., Lazarides A.A. (2005). Sensitivity of Metal Nanoparticle Surface Plasmon Resonance to the Dielectric Environment. J. Phys. Chem. B.

[58] Jain P.K., Eustis S., El-Sayed M.A. (2006). Plasmon Coupling in Nanorod Assemblies: Optical Absorption, Discrete Dipole Approximation Simulation, and Exciton-Coupling Model. J. Phys. Chem. B.

[59] Cattoni A., Ghenuche P., Haghiri-Gosnet A.-M., Decanini D., Chen J., Pelouard J.-L., Collin S. (2011). λ^3^/1000 Plasmonic Nanocavities for Biosensing Fabricated by Soft UV Nanoimprint Lithography. Nano Lett..

[60] Hong Y.A., Ha J.W. (2022). Enhanced refractive index sensitivity of localized surface plasmon resonance inflection points in single hollow gold nanospheres with inner cavity. Sci. Rep..

[61] Yang X., Sun Z., Low T., Hu H., Guo X., de Abajo F.J.G., Avouris P., Dai Q. (2018). Nanomaterial-Based Plasmon-Enhanced Infrared Spectroscopy. Adv. Mater..

[62] Sharifi M., Attar F., Saboury A.A., Akhtari K., Hooshmand N., Hasan A., El-Sayed M.A., Falahati M. (2019). Plasmonic gold nanoparticles: Optical manipulation, imaging, drug delivery and therapy. J. Control. Release.

[63] Hoa X., Kirk A., Tabrizian M. (2007). Towards integrated and sensitive surface plasmon resonance biosensors: A review of recent progress. Biosens. Bioelectron..

[64] Loiseau A., Asila V., Boitel-Aullen G., Lam M., Salmain M., Boujday S. (2019). Silver-Based Plasmonic Nanoparticles for and Their Use in Biosensing. Biosensors.

[65] Gawande M.B., Goswami A., Felpin F.-X., Asefa T., Huang X., Silva R., Zou X., Zboril R., Varma R.S. (2016). Cu and Cu-Based Nanoparticles: Synthesis and Applications in Catalysis. Chem. Rev..

[66] Rifat A.A., Mahdiraji G.A., Ahmed R., Chow D.M., Sua Y.M., Shee Y.G., Adikan F.R.M. (2015). Copper-Graphene-Based Photonic Crystal Fiber Plasmonic Biosensor. IEEE Photon- J..

[67] Zheng B.Y., Wang Y., Nordlander P., Halas N.J. (2014). Color-Selective and CMOS-Compatible Photodetection Based on Aluminum Plasmonics. Adv. Mater..

[68] Knight M.W., Liu L., Wang Y., Brown L., Mukherjee S., King N.S., Everitt H.O., Nordlander P., Halas N.J. (2012). Aluminum Plasmonic Nanoantennas. Nano Lett..

[69] McPeak K.M., Jayanti S.V., Kress S.J.P., Meyer S., Iotti S., Rossinelli A., Norris D.J. (2015). Plasmonic Films Can Easily Be Better: Rules and Recipes. ACS Photonics.

[70] Kim H., Osofsky M., Prokes S.M., Glembocki O.J., Piqué A. (2013). Optimization of Al-doped ZnO films for low loss plasmonic materials at telecommunication wavelengths. Appl. Phys. Lett..

[71] Taliercio T., Biagioni P. (2019). Semiconductor infrared plasmonics. Nanophotonics.

[72] Guler U., Shalaev V.M., Boltasseva A. (2015). Nanoparticle plasmonics: Going practical with transition metal nitrides. Mater. Today.

[73] Saha S., Dutta A., Kinsey N., Kildishev A.V., Shalaev V.M., Boltasseva A. (2018). On-Chip Hybrid Photonic-Plasmonic Waveguides with Ultrathin Titanium Nitride Films. ACS Photonics.

[74] Bao Q., Loh K.P. (2012). Graphene Photonics, Plasmonics, and Broadband Optoelectronic Devices. ACS Nano.

[75] Otsuji T., Popov V., Ryzhii V. (2014). Active graphene plasmonics for terahertz device applications. J. Phys. D Appl. Phys..

[76] Chen Y.-W., Su Y.-L., Hu S.-H., Chen S.-Y. (2016). Functionalized graphene nanocomposites for enhancing photothermal therapy in tumor treatment. Adv. Drug Deliv. Rev..

[77] Altug H., Oh S.-H., Maier S.A., Homola J. (2022). Advances and applications of nanophotonic biosensors. Nat. Nanotechnol..

[78] Hong G., Li C., Qi L. (2010). Facile Fabrication of Two-Dimensionally Ordered Macroporous Silver Thin Films and Their Application in Molecular Sensing. Adv. Funct. Mater..

[79] Xia Y., Ye J., Tan K., Wang J., Yang G. (2013). Colorimetric Visualization of Glucose at the Submicromole Level in Serum by a Homogenous Silver Nanoprism–Glucose Oxidase System. Anal. Chem..

[80] Dong C., Zhang X., Cai H., Cao C. (2016). Green synthesis of biocompatible silver nanoparticles mediated by Osmanthus fragrans extract in aqueous solution. Optik.

[81] Sun L., Zhang C., Wang C.-Y., Su P.-H., Zhang M., Gwo S., Shih C.-K., Li X., Wu Y. (2017). Enhancement of Plasmonic Performance in Epitaxial Silver at Low Temperature. Sci. Rep..

[82] Alkilany A.M., Lohse S.E., Murphy C.J. (2013). The Gold Standard: Gold Nanoparticle Libraries To Understand the Nano–Bio Interface. Acc. Chem. Res..

[83] Amendola V., Pilot R., Frasconi M., Marago O.M., Iatì M.A. (2017). Surface plasmon resonance in gold nanoparticles: A review. J. Phys. Condens. Matter.

[84] Biju V. (2014). Chemical modifications and bioconjugate reactions of nanomaterials for sensing, imaging, drug delivery and therapy. Chem. Soc. Rev..

[85] Elahi N., Kamali M., Baghersad M.H. (2018). Recent biomedical applications of gold nanoparticles: A review. Talanta.

[86] Xia Y., Halas N.J. (2005). Shape-Controlled Synthesis and Surface Plasmonic Properties of Metallic Nanostructures. MRS Bull..

[87] Li N., Zhao P., Astruc D. (2014). Anisotropic Gold Nanoparticles: Synthesis, Properties, Applications, and Toxicity. Angew. Chem. Int. Ed..

[88] Dondapati S.K., Sau T.K., Hrelescu C., Klar T.A., Stefani F.D., Feldmann J. (2010). Label-free Biosensing Based on Single Gold Nanostars as Plasmonic Transducers. ACS Nano.

[89] Wang X.-H., Li Y., Wang H., Fu Q., Peng J., Wang Y., Du J., Zhou Y., Zhan L. (2010). Gold nanorod-based localized surface plasmon resonance biosensor for sensitive detection of hepatitis B virus in buffer, blood serum and plasma. Biosens. Bioelectron..

[90] Lee S.-W., Lee K.-S., Ahn J., Lee J.-J., Kim M.-G., Shin Y.-B. (2011). Highly Sensitive Biosensing Using Arrays of Plasmonic Au Nanodisks Realized by Nanoimprint Lithography. ACS Nano.

[91] Jain P.K., Lee K.S., El-Sayed I.H., El-Sayed M.A. (2006). Calculated Absorption and Scattering Properties of Gold Nanoparticles of Different Size, Shape, and Composition: Applications in Biological Imaging and Biomedicine. J. Phys. Chem. B.

[92] Tian L., Liu K.-K., Morrissey J.J., Gandra N., Kharasch E.D., Singamaneni S. (2014). Gold nanocages with built-in artificial antibodies for label-free plasmonic biosensing. J. Mater. Chem. B.

[93] Li X., Soler M., Özdemir C.I., Belushkin A., Yesilköy F., Altug H. (2017). Plasmonic nanohole array biosensor for label-free and real-time analysis of live cell secretion. Lab A Chip.

[94] Brolo A.G. (2012). Plasmonics for future biosensors. Nat. Photonics.

[95] Li J., Cushing S., Zheng P., Meng F., Chu D., Wu N. (2013). Plasmon-induced photonic and energy-transfer enhancement of solar water splitting by a hematite nanorod array. Nat. Commun..

[96] Cetin A.E., Coskun A., Galarreta B., Huang M., Herman D., Ozcan A., Altug H. (2014). Handheld high-throughput plasmonic biosensor using computational on-chip imaging. Light Sci. Appl..

[97] Vestri A., Rippa M., Marchesano V., Sagnelli D., Margheri G., Zhou J., Petti L. (2021). LSPR immuno-sensing based on iso-Y nanopillars for highly sensitive and specific imidacloprid detection. J. Mater. Chem. B.

[98] Vogel N., Zieleniecki J., Köper I. (2012). As flat as it gets: Ultrasmooth surfaces from template-stripping procedures. Nanoscale.

[99] Weiss N.O., Zhou H., Liao L., Liu Y., Jiang S., Huang Y., Duan X. (2012). Graphene: An Emerging Electronic Material. Adv. Mater..

[100] Colas F., Barchiesi D., Kessentini S., Toury T., De La Chapelle M.L. (2015). Comparison of adhesion layers of gold on silicate glasses for SERS detection. J. Opt..

[101] Knight M.W., King N.S., Liu L., Everitt H.O., Nordlander P., Halas N.J. (2014). Aluminum for Plasmonics. ACS Nano.

[102] McClain M.J., Schlather A.E., Ringe E., King N.S., Liu L., Manjavacas A., Knight M.W., Kumar I., Whitmire K.H., Everitt H.O. (2015). Aluminum Nanocrystals. Nano Lett..

[103] Ekinci Y., Solak H.H., Löffler J.F. (2008). Plasmon resonances of aluminum nanoparticles and nanorods. J. Appl. Phys..

[104] Li W., Zhang L., Zhou J., Wu H. (2015). Well-designed metal nanostructured arrays for label-free plasmonic biosensing. J. Mater. Chem. C.

[105] Canalejas-Tejero V., Herranz S., Bellingham A., Moreno-Bondi M.C., Barrios C.A. (2014). Passivated Aluminum Nanohole Arrays for Label-Free Biosensing Applications. ACS Appl. Mater. Interfaces.

[106] Lee M., Kim J.U., Lee K.J., Ahn S., Shin Y.-B., Shin J., Park C.B. (2015). Aluminum Nanoarrays for Plasmon-Enhanced Light Harvesting. ACS Nano.

[107] Fedyanin D., Yakubovsky D.I., Kirtaev R., Volkov V.S. (2016). Ultralow-Loss CMOS Copper Plasmonic Waveguides. Nano Lett..

[108] Stebunov Y.V., Yakubovsky D.I., Fedyanin D.Y., Arsenin A.V., Volkov V.S. (2018). Superior Sensitivity of Copper-Based Plasmonic Biosensors. Langmuir.

[109] Zhou J., Wang Y., Zhang L., Li X. (2018). Plasmonic biosensing based on non-noble-metal materials. Chin. Chem. Lett..

[110] Wang C., Dai C., Hu Z., Li H., Yu L., Lin H., Bai J., Chen Y. (2019). Photonic cancer nanomedicine using the near infrared-II biowindow enabled by biocompatible titanium nitride nanoplatforms. Nanoscale Horiz..

[111] Dong S., Chen X., Gu L., Zhang L., Zhou X., Liu Z., Han P., Xu H., Yao J., Zhang X. (2011). A biocompatible titanium nitride nanorods derived nanostructured electrode for biosensing and bioelectrochemical energy conversion. Biosens. Bioelectron..

[112] Briggs J.A., Naik G.V., Petach T.A., Baum B.K., Goldhaber-Gordon D., Dionne J.A. (2016). Fully CMOS-compatible titanium nitride nanoantennas. Appl. Phys. Lett..

[113] Kinsey N., Ferrera M., Naik G.V., Babicheva V., Shalaev V.M., Boltasseva A. (2014). Experimental demonstration of titanium nitride plasmonic interconnects. Opt. Express.

[114] Gosciniak J., Atar F.B., Corbett B., Rasras M. (2019). CMOS-Compatible Titanium Nitride for On-Chip Plasmonic Schottky Photodetectors. ACS Omega.

[115] Lalisse A., Tessier G., Plain J., Baffou G. (2016). Plasmonic efficiencies of nanoparticles made of metal nitrides (TiN, ZrN) compared with gold. Sci. Rep..

[116] Zgrabik C.M., Hu E.L. (2015). Optimization of sputtered titanium nitride as a tunable metal for plasmonic applications. Opt. Mater. Express.

[117] Naik G.V., Schroeder J.L., Ni X., Kildishev A., Sands T., Boltasseva A. (2012). Titanium nitride as a plasmonic material for visible and near-infrared wavelengths. Opt. Mater. Express.

[118] Monfared Y.E. (2020). Overview of Recent Advances in the Design of Plasmonic Fiber-Optic Biosensors. Biosensors.

[119] Monfared Y.E. (2020). Refractive Index Sensor Based on Surface Plasmon Resonance Excitation in a D-Shaped Photonic Crystal Fiber Coated by Titanium Nitride. Plasmonics.

[120] Kaur V., Singh S. (2019). Design of titanium nitride coated PCF-SPR sensor for liquid sensing applications. Opt. Fiber Technol..

[121] Rifat A.A., Mahdiraji G.A., Sua Y.M., Ahmed R., Shee Y.G., Adikan F.R.M. (2016). Highly sensitive multi-core flat fiber surface plasmon resonance refractive index sensor. Opt. Express.

[122] Shkondin E., Repän T., Takayama O., Lavrinenko A.V. (2017). High aspect ratio titanium nitride trench structures as plasmonic biosensor. Opt. Mater. Express.

[123] Qiu G., Ng S.P., Wu C.-M.L. (2018). Label-free surface plasmon resonance biosensing with titanium nitride thin film. Biosens. Bioelectron..

[124] Popov A., Tselikov G., Dumas N., Berard C., Metwally K., Jones N., Al-Kattan A., Larrat B., Braguer D., Mensah S. (2019). Laser- synthesized TiN nanoparticles as promising plasmonic alternative for biomedical applications. Sci. Rep..

[125] Cortie M.B., Giddings J., Dowd A. (2010). Optical properties and plasmon resonances of titanium nitride nanostructures. Nanotechnology.

[126] Guler U., Ndukaife J.C., Naik G.V., Nnanna A.G.A., Kildishev A.V., Shalaev V.M., Boltasseva A. (2013). Local Heating with Lithographically Fabricated Plasmonic Titanium Nitride Nanoparticles. Nano Lett..

[127] Barho F.B., Gonzalez-Posada F., Milla M.-J., Bomers M., Cerutti L., Tournie E., Taliercio T. (2018). Highly doped semiconductor plasmonic nanoantenna arrays for polarization selective broadband surface-enhanced infrared absorption spectroscopy of vanillin. Nanophotonics.

[128] Kriegel I., Scotognella F., Manna L. (2017). Plasmonic doped semiconductor nanocrystals: Properties, fabrication, applications and perspectives. Phys. Rep..

[129] Faucheaux J.A., Stanton A.L.D., Jain P.K. (2014). Plasmon Resonances of Semiconductor Nanocrystals: Physical Principles and New Opportunities. J. Phys. Chem. Lett..

[130] Yang L., Peng Y., Yang Y., Liu J., Huang H., Yu B., Zhao J., Lu Y., Huang Z., Li Z. (2019). A Novel Ultra-Sensitive Semiconductor SERS Substrate Boosted by the Coupled Resonance Effect. Adv. Sci..

[131] Li D., Ning C.Z. (2011). All-semiconductor active plasmonic system in mid-infrared wavelengths. Opt. Express.

[132] Panah M.E.A., Han L., Norrman K., Pryds N., Nadtochiy A., Zhukov A., Lavrinenko A.V., Semenova E.S. (2017). Mid-IR optical properties of silicon doped InP. Opt. Mater. Express.

[133] Velický M., Toth P.S. (2017). From two-dimensional materials to their heterostructures: An electrochemist’s perspective. Appl. Mater. Today.

[134] Kalantar-Zadeh K., Ou J.Z., Daeneke T., Strano M.S., Pumera M., Gras S. (2015). Two-Dimensional Transition Metal Dichalcogenides in Biosystems. Adv. Funct. Mater..

[135] Zhang N.M.Y., Li K., Zhang T., Shum P., Wang Z., Wang Z., Zhang N., Wu T., Wei L. (2018). Electron-Rich Two-Dimensional Molybdenum Trioxides for Highly Integrated Plasmonic Biosensing. ACS Photonics.

[136] Nalwa H.S. (2020). A review of molybdenum disulfide (MoS_2_) based photodetectors: From ultra-broadband, self-powered to flexible devices. RSC Adv..

[137] Wang J., Fang H., Wang X., Chen X., Lu W., Hu W. (2017). Recent Progress on Localized Field Enhanced Two-dimensional Material Photodetectors from Ultraviolet-Visible to Infrared. Small.

[138] Lee G., Kim S., Jung S., Jang S., Kim J. (2017). Suspended black phosphorus nanosheet gas sensors. Sens. Actuators B Chem..

[139] Lei W., Liu G., Zhang J., Liu M. (2017). Black phosphorus nanostructures: Recent advances in hybridization, doping and functionalization. Chem. Soc. Rev..

[140] Fang Y., Ge Y., Wang C., Zhang H. (2020). Mid-Infrared Photonics Using 2D Materials: Status and Challenges. Laser Photonics Rev..

[141] Avani A., Anila E. (2022). Recent advances of MoO_3_ based materials in energy catalysis: Applications in hydrogen evolution and oxygen evolution reactions. Int. J. Hydrog. Energy.

[142] Zhang W.-B., Qu Q., Lai K. (2017). High-Mobility Transport Anisotropy in Few-Layer MoO_3_ and Its Origin. ACS Appl. Mater. Interfaces.

[143] Liu Q., Wu Y., Zhang J., Chen K., Huang C., Chen H., Qiu X. (2019). Plasmonic MoO_3_-x nanosheets with tunable oxygen vacancies as efficient visible light responsive photocatalyst. Appl. Surf. Sci..

[144] Rodrigo D., Limaj O., Janner D., Etezadi D., García de Abajo F.J., Pruneri V., Altug H. (2015). Mid-infrared plasmonic biosensing with graphene. Science.

[145] Rodrigo D., Tittl A., Limaj O., de Abajo F.J.G., Pruneri V., Altug H. (2017). Double-layer graphene for enhanced tunable infrared plasmonics. Light Sci. Appl..

[146] Zeng S., Sreekanth K.V., Shang J., Yu T., Chen C.-K., Yin F., Baillargeat D., Coquet P., Ho H.-P., Kabashin A.V. (2015). Graphene-Gold Metasurface Architectures for Ultrasensitive Plasmonic Biosensing. Adv. Mater..

[147] Ouyang Q., Zeng S., Jiang L., Hong L., Xu G., Dinh X.Q., Qian J., He S., Qu J., Coquet P. (2016). Sensitivity Enhancement of Transition Metal Dichalcogenides/Silicon Nanostructure-based Surface Plasmon Resonance Biosensor. Sci. Rep..

[148] Cheng H., Wen M., Ma X., Kuwahara Y., Mori K., Dai Y., Huang B., Yamashita H. (2016). Hydrogen Doped Metal Oxide Semiconductors with Exceptional and Tunable Localized Surface Plasmon Resonances. J. Am. Chem. Soc..

[149] Wu L., Chu H.S., Koh W.S., Li E.P. (2010). Highly sensitive graphene biosensors based on surface plasmon resonance. Opt. Express.

[150] Salihoglu O., Balci S., Kocabas C. (2012). Plasmon-polaritons on graphene-metal surface and their use in biosensors. Appl. Phys. Lett..

[151] Verma A., Prakash A., Tripathi R. (2015). Sensitivity enhancement of surface plasmon resonance biosensor using graphene and air gap. Opt. Commun..

[152] De Abajo F.J.G. (2014). Graphene Plasmonics: Challenges and Opportunities. ACS Photonics.

[153] Kim J.H., Jin H.M., Yang G.G., Han K.H., Yun T., Shin J.Y., Jeong S.-J., Kim S.O. (2020). Smart Nanostructured Materials based on Self-Assembly of Block Copolymers. Adv. Funct. Mater..

[154] Yang Y., Liu W., Lin Z., Pan R., Gu C., Li J. (2021). Plasmonic hybrids of two-dimensional transition metal dichalcogenides and nanoscale metals: Architectures, enhanced optical properties and devices. Mater. Today Phys..

[155] Yuan Y., Yu X., Ouyang Q., Shao Y., Song J., Qu J., Yong K.-T. (2018). Highly anisotropic black phosphorous-graphene hybrid architecture for ultrassensitive plasmonic biosensing: Theoretical insight. 2D Mater..

[156] Zeng Y., Guo Z. (2021). Synthesis and stabilization of black phosphorus and phosphorene: Recent progress and perspectives. iScience.

[157] Zhou Q., Chen Q., Tong Y., Wang J. (2016). Light-Induced Ambient Degradation of Few-Layer Black Phosphorus: Mechanism and Protection. Angew. Chem. Int. Ed..

[158] Fan M., Andrade G.F., Brolo A.G. (2011). A review on the fabrication of substrates for surface enhanced Raman spectroscopy and their applications in analytical chemistry. Anal. Chim. Acta.

[159] Khan S., Ali S., Bermak A. (2019). Recent Developments in Printing Flexible and Wearable Sensing Electronics for Healthcare Applications. Sensors.

[160] López-Muñoz G.A., Estévez M., Vázquez-García M., Berenguel-Alonso M., Alonso-Chamarro J., Homs-Corbera A., Lechuga L.M. (2018). Gold/silver/gold trilayer films on nanostructured polycarbonate substrates for direct and label-free nanoplasmonic biosensing. J. Biophotonics.

[161] Qin Y., Howlader M.M., Deen M.J., Haddara Y.M., Selvaganapathy P.R. (2014). Polymer integration for packaging of implantable sensors. Sens. Actuators B Chem..

[162] Wen J., Zhang H., Chen H., Zhang W., Chen J. (2015). Stretchable plasmonic substrate with tunable resonances for surface-enhanced Raman spectroscopy. J. Opt..

[163] Mou L., Jiang X. (2017). Materials for Microfluidic Immunoassays: A Review. Adv. Health Mater..

[164] Torino S., Conte L., Iodice M., Coppola G., Prien R.D. (2017). PDMS membranes as sensing element in optical sensors for gas detection in water. Sens. Bio-Sens. Res..

[165] Jankauskaitė V., Narmontas P., Lazauskas A. (2019). Control of Polydimethylsiloxane Surface Hydrophobicity by Plasma Polymerized Hexamethyldisilazane Deposition. Coatings.

[166] Bosq N., Guigo N., Persello J., Sbirrazzuoli N. (2014). Melt and glass crystallization of PDMS and PDMS silica nanocomposites. Phys. Chem. Chem. Phys..

[167] Popok V.N., Hanif M., Mackova A., Mikšovå R. (2015). Structure and plasmonic properties of thin PMMA layers with ion-synthesized Ag nanoparticles. J. Polym. Sci. Part B Polym. Phys..

[168] Vodišek N., Šuligoj A., Korte D., Štangar U.L. (2018). Transparent Photocatalytic Thin Films on Flexible Polymer Substrates. Materials.

[169] Fan X., Nie W., Tsai H., Wang N., Huang H., Cheng Y., Wen R., Ma L., Yan F., Xia Y. (2019). PEDOT: PSS for Flexible and Stretchable Electronics: Modifications, Strategies, and Applications. Adv. Sci..

[170] Xu J., Wang S., Wang G.-J.N., Zhu C., Luo S., Jin L., Gu X., Chen S., Feig V.R., To J.W.F. (2017). Highly stretchable polymer semiconductor films through the nanoconfinement effect. Science.

[171] Lee S.H., Sohn J.S., Kulkarni S.B., Patil U.M., Jun S.C., Kim J.H. (2014). Modified physico–chemical properties and supercapacitive performance via DMSO inducement to PEDOT: PSS active layer. Org. Electron..

[172] Liu Y., Chen Q., Du X., Li L., Li P. (2018). Surface modification of polyethylene terephthalate films by direct fluorination. AIP Adv..

[173] Kawai F., Kawabata T., Oda M. (2019). Current knowledge on enzymatic PET degradation and its possible application to waste stream management and other fields. Appl. Microbiol. Biotechnol..

[174] Tsao C.-W. (2016). Polymer Microfluidics: Simple, Low-Cost Fabrication Process Bridging Academic Lab Research to Commercialized Production. Micromachines.

[175] Aćimović S.S., Ortega M.A., Sanz V., Berthelot J., Garcia-Cordero J.L., Renger J., Maerkl S.J., Kreuzer M.P., Quidant R. (2014). LSPR Chip for Parallel, Rapid, and Sensitive Detection of Cancer Markers in Serum. Nano Lett..

[176] Bomers M., Charlot B., Barho F., Chanuel A., Mezy A., Cerutti L., Gonzalez-Posada F., Taliercio T. (2020). Microfluidic surface-enhanced infrared spectroscopy with semiconductor plasmonics for the fingerprint region. React. Chem. Eng..

[177] Woo S., Jeong J.H., Lyu H.K., Han Y.S., Kim Y. (2012). In situ-prepared composite materials of PEDOT: PSS buffer layer-metal nanoparticles and their application to organic solar cells. Nanoscale Res. Lett..

[178] Mori T., Mori T., Tanaka Y., Suzaki Y., Yamaguchi K. (2017). Fabrication of single-crystalline plasmonic nanostructures on transparent and flexible amorphous substrates. Sci. Rep..

[179] Halldorsson S., Lucumi E., Gómez-Sjöberg R., Fleming R.M. (2015). Advantages and challenges of microfluidic cell culture in polydimethylsiloxane devices. Biosens. Bioelectron..

[180] Akther F., Yakob S.B., Nguyen N.T., Ta H.T. (2020). Surface modification techniques for endothelial cell seeding in PDMS microfluidic devices. Biosensors.

[181] Li D., Lai W.-Y., Zhang Y.-Z., Huang W. (2018). Printable Transparent Conductive Films for Flexible Electronics. Adv. Mater..

[182] Kayser L.V., Lipomi D.J. (2019). Stretchable Conductive Polymers and Composites Based on PEDOT and PEDOT: PSS. Adv. Mater..

[183] Jiang Y., Xu M., Yadavalli V.K. (2019). Silk Fibroin-Sheathed Conducting Polymer Wires as Organic Connectors for Biosensors. Biosensors.

[184] Bihar E., Wustoni S., Pappa A.-M., Salama K.N., Baran D., Inal S. (2018). A fully inkjet-printed disposable glucose sensor on paper. Npj Flex. Electron..

[185] Chou J.-A., Chung C.-L., Ho P.-C., Luo C.-H., Tsai Y.-H., Wu C.-K., Kuo C.-W., Hsiao Y.-S., Yu H.-H., Chen P. (2019). Organic Electrochemical Transistors/SERS-Active Hybrid Biosensors Featuring Gold Nanoparticles Immobilized on Thiol-Functionalized PEDOT Films. Front. Chem..

[186] Notarianni M., Vernon K., Chou A., Aljada M., Liu J., Motta N. (2014). Plasmonic effect of gold nanoparticles in organic solar cells. Sol. Energy.

[187] Leordean C., Gabudean A.-M., Canpean V., Astilean S. (2013). Easy and cheap fabrication of ordered pyramidal-shaped plasmonic substrates for detection and quantitative analysis using surface-enhanced Raman spectroscopy. Analyst.

[188] Cheng C., Xu X., Lei H., Li B. (2016). Plasmon-assisted trapping of nanoparticles using a silver-nanowire-embedded PMMA nanofiber. Sci. Rep..

[189] Alsawafta M., Badilescu S., Paneri A., Truong V.-V., Packirisamy M. (2011). Gold-Poly (methyl methacrylate) Nanocomposite Films for Plasmonic Biosensing Applications. Polymers.

[190] Nichols S.P., Koh A., Storm W.L., Shin J.H., Schoenfisch M.H. (2013). Biocompatible Materials for Continuous Glucose Monitoring Devices. Chem. Rev..

[191] Fan M., Thompson M., Andrade M.L., Brolo A.G. (2010). Silver Nanoparticles on a Plastic Platform for Localized Surface Plasmon Resonance Biosensing. Anal. Chem..

[192] Xu M., Obodo D., Yadavalli V.K. (2019). The design, fabrication, and applications of flexible biosensing devices. Biosens. Bioelectron..

[193] Jiang Y., Liang Y., Zhang H., Zhang W., Tu S. (2014). Preparation and biocompatibility of grafted functional β-cyclodextrin copolymers from the surface of PET films. Mater. Sci. Eng. C.

[194] Ding Y., Duan Y., Huang Y. (2015). Electrohydrodynamically Printed, Flexible Energy Harvester Using In Situ Poled Piezoelectric Nanofibers. Energy Technol..

[195] Nishiguchi K., Sueyoshi K., Hisamoto H., Endo T. (2016). Fabrication of gold-deposited plasmonic crystal based on nanoimprint lithography for label-free biosensing application. Jpn. J. Appl. Phys..

[196] Pandiyaraj K.N., Selvarajan V., Deshmukh R. (2010). Effects of operating parameters on DC glow discharge plasma induced PET film surface. J. Phys. Conf. Ser..

[197] Huang Q., Devetter B.M., Roosendaal T., LaBerge M., Bernacki B.E., Alvine K.J. (2017). Fabrication of large area flexible nanoplasmonic templates with flow coating. Rev. Sci. Instrum..

[198] Park S.-G., Mun C., Xiao X., Braun A., Kim S., Giannini V., Maier S.A., Kim D.-H. (2017). Surface Energy-Controlled SERS Substrates for Molecular Concentration at Plasmonic Nanogaps. Adv. Funct. Mater..

[199] Moreno I., Navascués N., Arruebo M., Irusta S., Santamaria J. (2013). Facile preparation of transparent and conductive polymer films based on silver nanowire/polycarbonate nanocomposites. Nanotechnology.

[200] Liu L., Zhang Q., Lu Y., Du W., Li B., Cui Y., Yuan C., Zhan P., Ge H., Wang Z. (2017). A high-performance and low cost SERS substrate of plasmonic nanopillars on plastic film fabricated by nanoimprint lithography with AAO template. AIP Adv..

[201] Li Y., Wang Z., Ou L.M.L., Yu H.-Z. (2007). DNA Detection on Plastic: Surface Activation Protocol to Convert Polycarbonate Substrates to Biochip Platforms. Anal. Chem..

[202] Lee K.-L., Chen P.-W., Wu S.-H., Huang J.-B., Yang S.-Y., Wei P.-K. (2012). Enhancing Surface Plasmon Detection Using Template-Stripped Gold Nanoslit Arrays on Plastic Films. ACS Nano.

[203] Jankowski P., Ogonczyk D., Kosinski A., Lisowski W., Garstecki P. (2011). Hydrophobic modification of polycarbonate for reproducible and stable formation of biocompatible microparticles. Lab A Chip.

[204] Schulz U., Lau K., Kaiser N. (2008). Antireflection coating with UV-protective properties for polycarbonate. Appl. Opt..

[205] Kang M.S., Lee S.Y., Kim K.S., Han D.-W. (2020). State of the Art Biocompatible Gold Nanoparticles for Cancer Theragnosis. Pharmaceutics.

[206] Willets K.A., Van Duyne R.P. (2007). Localized Surface Plasmon Resonance Spectroscopy and Sensing. Annu. Rev. Phys. Chem..

[207] López-Muñoz G.A., Estevez M.-C., Peláez-Gutierrez E.C., Homs-Corbera A., García-Hernandez M.C., Imbaud J.I., Lechuga L.M. (2017). A label-free nanostructured plasmonic biosensor based on Blu-ray discs with integrated microfluidics for sensitive biodetection. Biosens. Bioelectron..

[208] Alharbi R., Irannejad M., Yavuz M. (2019). A Short Review on the Role of the Metal-Graphene Hybrid Nanostructure in Promoting the Localized Surface Plasmon Resonance Sensor Performance. Sensors.

[209] Liu K., Li N., Sadana D.K., Sorger V.J. (2016). Integrated Nanocavity Plasmon Light Sources for On-Chip Optical Interconnects. ACS Photonics.

[210] Jiao F., Li F., Shen J., Guan C., Khan S.A., Wang J., Yang Z., Zhu J. (2021). Wafer-scale flexible plasmonic metasurface with passivated aluminum nanopillars for high-sensitivity immunosensors. Sens. Actuators B Chem..

[211] Spitzberg J.D., Zrehen A., Van Kooten X.F., Meller A. (2019). Plasmonic-Nanopore Biosensors for Superior Single-Molecule Detection. Adv. Mater..

[212] Ma X., Sim S.J. (2020). Single plasmonic nanostructures for biomedical diagnosis. J. Mater. Chem. B.

[213] Xu K., Zhou R., Takei K., Hong M. (2019). Toward Flexible Surface-Enhanced Raman Scattering (SERS) Sensors for Point-of-Care Diagnostics. Adv. Sci..

[214] Liu W., Zhuo Q., Wen K., Zou Q., Hu X., Qin Y. (2021). Integrated plasmonic biosensor on a vertical cavity surface emitting laser platform. Opt. Express.

[215] Hang Y., Boryczka J., Wu N. (2021). Visible-light and near-infrared fluorescence and surface-enhanced Raman scattering point-of-care sensing and bio-imaging: A review. Chem. Soc. Rev..

[216] Soler M., Lechuga L.M. (2021). Principles, technologies, and applications of plasmonic biosensors. J. Appl. Phys..

[217] Thrift W.J., Cabuslay A., Laird A.B., Ranjbar S., Hochbaum A.I., Ragan R. (2019). Surface-Enhanced Raman Scattering-Based Odor Compass: Locating Multiple Chemical Sources and Pathogens. ACS Sens..

[218] Cui F., Yue Y., Zhang Y., Zhang Z., Zhou H.S. (2020). Advancing Biosensors with Machine Learning. ACS Sens..

[219] Plou J., Valera P.S., García I., de Albuquerque C.D.L., Carracedo A., Liz-Marzán L.M. (2022). Prospects of Surface-Enhanced Raman Spectroscopy for Biomarker Monitoring toward Precision Medicine. ACS Photonics.

